# Forward Genetics Approach Reveals Host Genotype-Dependent Importance of Accessory Chromosomes in the Fungal Wheat Pathogen *Zymoseptoria tritici*

**DOI:** 10.1128/mBio.01919-17

**Published:** 2017-11-28

**Authors:** Michael Habig, Jakob Quade, Eva Holtgrewe Stukenbrock

**Affiliations:** Department of Environmental Genomics, Christian-Albrechts-University of Kiel and Max Planck Institute for Evolutionary Biology, Plön, Germany; University of Minnesota; Tel Aviv University

**Keywords:** genome plasticity, functional dissection, host-pathogen interactions, induced chromosome deletions, virulence

## Abstract

The fungal wheat pathogen *Zymoseptoria tritici* possesses a large complement of accessory chromosomes showing presence/absence polymorphism among isolates. These chromosomes encode hundreds of genes; however, their functional role and why the chromosomes have been maintained over long evolutionary times are so far not known. In this study, we addressed the functional relevance of eight accessory chromosomes in reference isolate IPO323. We induced chromosome losses by inhibiting the β-tubulin assembly during mitosis using carbendazim and generated several independent isogenic strains, each lacking one of the accessory chromosomes. We confirmed chromosome losses by electrophoretic karyotyping and whole-genome sequencing. To assess the importance of the individual chromosomes during host infection, we performed *in planta* assays comparing disease development results in wild-type and chromosome mutant strains. Loss of the accessory chromosomes 14, 16, 18, 19, and 21 resulted in increased virulence on wheat cultivar Runal but not on cultivars Obelisk, Titlis, and Riband. Moreover, some accessory chromosomes affected the switch from biotrophy to necrotrophy as strains lacking accessory chromosomes 14, 18, 19, and 21 showed a significantly earlier onset of necrosis than the wild type on the Runal cultivar. In general, we observed that the timing of the lifestyle switch affects the fitness of *Z. tritici*. Taking the results together, this study was the first to use a forward-genetics approach to demonstrate a cultivar-dependent functional relevance of the accessory chromosomes of *Z. tritici* during host infection.

## IMPORTANCE

*Zymoseptoria tritici* is a prominent fungal pathogen of wheat of worldwide distribution. This fungus shows a remarkable genome organization, with a large number of chromosomes that are present in only some isolates and therefore considered to be “accessory” chromosomes. To date, the function of these accessory chromosomes in *Z. tritici* has been unknown, although their maintenance in the species over evolutionary times suggests a functional relevance. Here we deleted whole accessory chromosomes to test the effect of these chromosomes on host specificity and virulence of the fungus. We show for the first time that some accessory chromosomes of *Z. tritici* affect the fitness of the fungus during host infection in a cultivar-dependent manner. These results show that the accessory chromosomes encode host-specific virulence determinants having a negative effect on fitness. Understanding the population dynamic of the accessory chromosomes and the molecular interaction of pathogen and plant traits is crucial to improve wheat-breeding strategies.

## INTRODUCTION

The fungus *Zymoseptoria tritici* (synonym *Mycosphaerella graminicola*) is the causative agent of the wheat disease Septoria tritici blotch. The pathogen is distributed worldwide where wheat is grown and greatly impacts crop yields. Experimental and genomic approaches have shed light on the host-associated lifecycle of *Z. tritici*, and yet the underlying determinants of pathogenicity and host specificity are largely unknown (see, e.g., references 1–4). The fungus establishes an intercellular hyphal network upon infection through open stomata. It propagates without causing disease symptoms during a biotrophic phase of 7 to 14 days. For asexual sporulation, *Z. tritici* induces host cell death allowing necrotrophic feeding and the development of pycnidia in substomatal cavities (5). Population genetic studies have confirmed that sexual mating occurs frequently in the field (6, 7), and yet the sexual stage and conditions required for sexual development are not known.

The haploid fungus is well suited for genetic and genomic studies as its genome is one of the best-assembled fungal genomes, with 21 fully sequenced chromosomes of the IPO323 reference isolate (1). These chromosomes can be classified into 13 core chromosomes common to all isolates and a set of supernumerary or accessory chromosomes showing presence/absence polymorphism among isolates (8). Reference strain IPO323 has eight accessory chromosomes, representing the highest number of reported accessory chromosomes in a filamentous fungus and comprising 12% of the genome of this isolate (1).

Accessory chromosomes have been described for many eukaryotes (see, e.g., references 9–11). Prominent examples among filamentous fungi include the plant-pathogenic species *Fusarium oxysporum*, *Nectria haematococca*, and *Leptosphaeria maculans*, in which accessory genomic elements were shown to contribute to host specificity and virulence (12–14). A similar role has not yet been described for the accessory chromosomes of *Z. tritici*. Similarly to reports in other fungi, the accessory chromosomes in *Z. tritici* vary in chromosomal organization from the core chromosomes by a lower gene density (1), a higher proportion of unique genes (15), and an enrichment in transposable elements (1). Recently, we were able to show that the telomeres, subtelomeric regions, and centromeres do not differ between accessory and core chromosomes and yet that the accessory chromosomes are greatly enriched in nucleosomes with H3K27 trimethylation (16), which is known to be associated with gene silencing (15). The accessory chromosomes are frequently lost during meiosis (1, 8, 17) and show a high level of structural variation, whereby isolates differ in the presence/absence of homologue chromosomes and can carry highly differentiated gene content due to numerous insertions and deletions (17–19). In the IPO323 reference isolate, more than 800 genes are located on the accessory chromosomes (20) and show, on average, lower expression than genes located on the core chromosomes during growth in axenic culture and infection of the wheat host (3, 15). However, among the genes located on the accessory chromosomes, 174 were classified as being highly expressed and 22 showed host-specific regulation (15). In a separate study, 79 of the genes located on the accessory chromosomes differed in expression during colonization and asexual reproduction (3). However, no functional analyses of these genes have been performed to date.

Intriguingly, synteny among regions of the accessory chromosome of *Z. tritici* and its sister species *Zymoseptoria ardabiliae* and *Zymoseptoria pseudotritici* indicates that the accessory chromosomes were inherited from a common ancestor and therefore appear to have been maintained since the speciation event ~11,000 years ago (21, 22). The accessory chromosomes evolve faster than the core chromosomes due to a relaxation of selection (23). Yet it is still unknown why the accessory chromosomes are maintained in the species. They were previously proposed to provide a repertoire of genes for adaptive evolution (24). Yet natural selection acts at the individual level, and a repertoire of genes would be advantageous only at the population level. Thus, we lack an evolutionary model to explain the maintenance of accessory chromosomes as a repertoire of genes for future generations of the pathogen. Alternatively, the accessory chromosomes may encode genes that provide a selective advantage only under specific conditions (11). In a heterogeneous environment, the accessory chromosomes may have been maintained by balancing selection, sometimes conferring a fitness advantage and sometimes not.

To understand the presence and evolutionary dynamics of these chromosomes, we need to determine their functional relevance. A functional genetic assessment should ideally use isogenic strains that differ only in the presence and absence of the gene or genetic element of interest. In the past, functional studies of the accessory chromosomes of *Z. tritici* relied on phenotyping of progenies with chromosome losses (8, 25). However, meiotic recombination additionally introduces variation throughout the genome of the progeny, making a comparison between accessory chromosome-containing and chromosome-lacking strains difficult. Such additional variation may be particularly strong in *Z. tritici* because of its high genomic plasticity, as highlighted in a recent study where more than 200 isolate-specific genes were described (19). Here, we circumvented these problems by inducing whole chromosome loss during mitosis to generate isogenic strains that then differed only in the presence/absence of an accessory chromosome. The fungicide carbendazim was used to manipulate mitosis and to disrupt β-tubulin assembly of the mitotic spindle (26). This approach allowed us to obtain at least one strain for each of the eight accessory chromosomes of the IPO323 reference isolate that had lost that one particular accessory chromosome. We demonstrate for the first time that accessory chromosomes of *Z. tritici* have a functional effect on the *in planta* phenotype of *Z. tritici* in a host cultivar-specific manner. In addition, we show that the wheat cultivar influences disease progression and that *Z. tritici* fitness is affected by the timing of the switch from biotrophy to necrotrophy. Taken together, these results provide the first evidence that a variety of host genotypes have different fitness effects on the accessory chromosomes in *Z. tritici* and may play a role in the distribution and dynamics of these small chromosomes.

## RESULTS

### Variation in the rate of chromosome losses among eight accessory chromosomes.

To induce aneuploidy and chromosome losses in *Z. tritici*, we treated cells with carbendazim during vegetative growth. In total, 188 independent liquid cultures of *Z. tritici* were inoculated in the presence and, as a control, in the absence of carbendazim for up to 2 weeks before being streaked onto yeast-malt-sucrose (YMS) (4 g/liter yeast extract, 4 g/liter malt extract, 4 g/liter sucrose) agar plates. We screened 2,892 colonies for the loss of accessory chromosomes by a multiplexed PCR targeting the right subtelomeric region of each of the eight accessory chromosomes (see Fig. S1 in the supplemental material). The loss of a chromosome was confirmed by at least three independent PCRs for each accessory chromosome, two targeting the left and right subtelomeric regions, respectively, and one targeting the centromeric region. In total, we could confirm 37 chromosome losses (relative frequency, 1.28%) by PCR screen (Table 1). The total number of colonies characterized using PCR-based karyotyping in the presence of carbendazim was 2,506 (∑, 33 chromosomal losses; relative frequency, 1.32%). The total number of colonies characterized using PCR-based karyotyping in the absence of carbendazim was 386 (∑, 4 chromosomal losses; relative frequency, 1.04%). Interestingly, the distribution of chromosome losses varied significantly among chromosomes (*P* = 0.009; chi-square test with Yates correction). A total of 13 *Z. tritici* strains had lost chromosome 15, while none lacked chromosome 19, suggesting different degrees of stability of the eight accessory chromosomes. Four chromosome losses were detected in control experiments without carbendazim (386 tested colonies). While the low number of chromosome losses in the nontreated strains prevented solid statistical inferences, we note that the frequency of spontaneous chromosomes losses did not deviate much from the frequency seen with carbendazim-treated strains.

10.1128/mBio.01919-17.2 FIG S1  Gel electrophoresis for PCR karyotyping of strains used in this study of (A) multiplexed PCR targeting the subtelomeric region of chromosomes 14 to 21 (B to I) by PCR (B) along chromosome 14, (C) along chromosome 15, (D) along chromosome 16, (E) along chromosome 17, (F) along chromosome 18, (G) along chromosome 19, (H) along chromosome 20, and (I) along chromosome 21. Download FIG S1, PDF file, 0.7 MB. Copyright © 2017 Habig et al. 2017 Habig et al. This content is distributed under the terms of the Creative Commons Attribution 4.0 International license.

**TABLE 1:** Absolute and relative frequencies of unique chromosomal loss events in IPO323 in the presence and absence of carbendazim

Accessory chromosome	No. of chromosomal losses		
	With carbendazim	Without carbendazim	Total
14	2	2	4
15	12	1	13
16	5	0	5
17	3	1	4
18	4	0	4
19	0	0	0
20	2	0	2
21	5	0	5

### Aneuploid strains are isogenic to IPO322.

**FIG 1:**
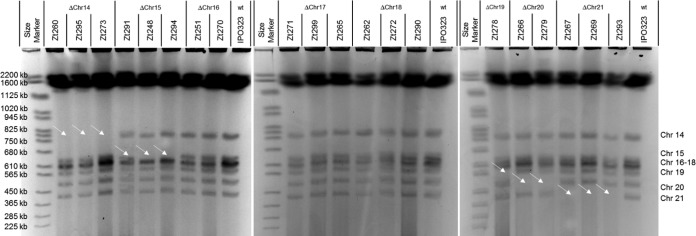
Pulsed-field gel electrophoresis for the accessory chromosomes of *Z. tritici*. Pictures of three pulsed-field gel electrophoresis gels for all strains used in this study are shown. Size marker, *Saccharomyces cerevisiae*. White arrows indicate missing chromosome bands. Absence of chromosome 14 is observed for strains Zt260, Zt295, and Zt273. Absence of chromosome 15 is observed for strains Zt291, Zt248, and Zt294; absence of chromosome 16 or 17 or 18 can be inferred for strains Zt251, Zt270, Zt271, Zt299, Zt265, Zt262, Zt272, and Zt290. Absence of chromosome 19 is observed for strain Zt278; absence of chromosome 20 is observed for strains Zt266 and Zt279; and absence of chromosome 21 is observed for strains Zt267, Zt269, and Zt293.

We applied two approaches to identify and exclude aneuploid strains that had acquired additional mutational changes, i.e., which did not remain isogenic to the progenitor strain IPO323. First, the chromosome sizes of all strains were analyzed using pulsed-field gel electrophoresis (PFGE). Please note that, in order to verify aneuploid strains that had lost one of each accessory chromosome of IPO323, we included the *Z. tritici* 278 (Zt278) strain derived from a separate study. Strain Zt278 was isolated from a pycnidium formed on a leaf infected with IPO323, and the strain was found to have lost accessory chromosome 19, which we could not delete with carbendazim *in vitro*. The PFGE analyses also allowed us to confirm the loss of accessory chromosomes 14, 15, 19, 20, and 21 for all the respective strains identified in the PCR assay (Fig. 1). Chromosomes 16, 17, and 18 could not be separated due to the small size differences (607 kb, 584 kb, and 574 kb); however, a lower intensity in the chromosome band correlated well with the expected chromosome loss for the respective strains (Fig. 1). Importantly, none of the strains showed any additional chromosomal aberration for accessory or core chromosomes (Fig. S2 and S3).

10.1128/mBio.01919-17.3 FIG S2  Pulsed-field gel electrophoresis for midsize core chromosomes of *Z. tritici*. Pictures of three pulsed-field gel electrophoresis gels for all strains used in this study are shown. Size marker, *Hansenula wingei* chromosomes. Download FIG S2, PDF file, 0.2 MB. Copyright © 2017 Habig et al. 2017 Habig et al. This content is distributed under the terms of the Creative Commons Attribution 4.0 International license.

10.1128/mBio.01919-17.4 FIG S3  Pulsed-field gel electrophoresis for large core chromosomes of *Z. tritici*. Pictures of three pulsed-field gel electrophoresis gels for all strains used in this study are shown. Size marker, *Schizosaccharomyces pombe* chromosomes. Download FIG S3, PDF file, 0.2 MB. Copyright © 2017 Habig et al. 2017 Habig et al. This content is distributed under the terms of the Creative Commons Attribution 4.0 International license.

**FIG 2:**
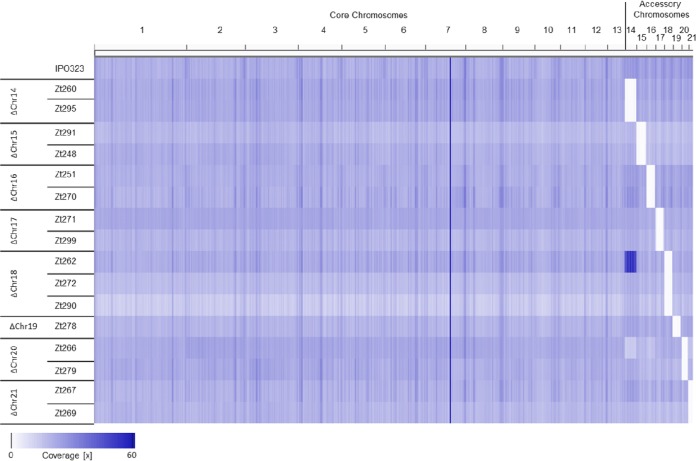
Genome-wide map of Illumina read coverage of the IPO323 progenitor strain and the aneuploid strains. The absence of reads mapped to the deleted accessory chromosomes confirms chromosome loss in the respective strains. Increased coverage of chromosome 14 in strain Zt262 indicates chromosome duplication, while the lower coverage of chromosome 14 in strain Zt266, on the other hand, suggests a mix of cells with and without this chromosome.

Second, we sequenced whole genomes for a subset of 16 strains, including the progenitor IPO323 strain, using next-generation sequencing technology. On average, 4 × 10^6^ paired-end Illumina reads were obtained for each strain, providing an average read coverage for all strains of 20× (see Table S1 in the supplemental material). Paired-end reads were mapped to the reference genome of IPO323 to identify rearrangements, deletions, duplications, single nucleotide polymorphisms (SNP), and indels. The resequencing confirmed the loss of accessory chromosomes in the tested aneuploid strains (Fig. 2). Furthermore, for 13 of the 16 resequenced strains, we did not find evidence of additional chromosomal rearrangements or deletions. Those 13 strains showed homogeneous read coverage on all core and accessory chromosomes comparable to the observed coverage for the resequenced IPO323 progenitor strain. Three strains, however, exhibited additional large-scale chromosomal variation in addition to the expected chromosome losses (Fig. 2). Strain Zt262 showed an increase in the read coverage for chromosome 14 consistent with a possible duplication of the chromosome. Strain Zt266 had reduced read coverage for chromosome 14, indicating that the sequenced strain comprised a mixture of cells with and without chromosome 14. For strain Zt248, we observed a deletion of a 6.6-kb fragment in the telomeric region of chromosome 14.

10.1128/mBio.01919-17.7 TABLE S1  Summary of next-generation sequencing data and read mapping to the IPO323 reference genome. Download TABLE S1, XLSX file, 0.01 MB. Copyright © 2017 Habig et al. 2017 Habig et al. This content is distributed under the terms of the Creative Commons Attribution 4.0 International license.

To further identify genomic changes in the aneuploid stains, we identified SNPs and small indels for all resequenced aneuploid strains using the genome of IPO323 as the reference (1). Resequencing of the IPO323 progenitor strain revealed 13 SNPs and two indels compared to the reference genome (Fig. S4). These novel SNPs either originated from sequencing errors in the Sanger-sequenced reference or in the Illumina-resequenced genome or represented mutations derived during laboratory propagation. An average of 3.2 SNP and indels per strain were novel and not present in the IPO323 progenitor (Table 2). Nine of these novel SNPs and indels were located in coding sequences in six strains (Zt248, Zt271, Zt272, Zt291, Zt295, and Zt299); seven of the SNP/indels in five strains (Zt248, Zt271, Zt272, Zt291, and Zt299) were nonsynonymous (Table S2). The remaining 10 strains did not contain any novel SNPs and indels in coding sequences, confirming that there were either no or very few genetic changes in the aneuploid *Z. tritici* strains beside the absence of the distinct accessory chromosomes.

10.1128/mBio.01919-17.5 FIG S4  Illustration of the locations of all single nucleotide polymorphisms (SNPs) and indels identified in all sequenced strains. Data represent genome-wide distribution of SNPs across the 13 core and 8 accessory chromosomes of reference strain IPO323. Download FIG S4, PDF file, 0.1 MB. Copyright © 2017 Habig et al. 2017 Habig et al. This content is distributed under the terms of the Creative Commons Attribution 4.0 International license.

10.1128/mBio.01919-17.8 TABLE S2  Summary of identified SNP and indels in the resequenced genomes of the aneuploid strains. Download TABLE S2, XLSX file, 0.01 MB. Copyright © 2017 Habig et al. 2017 Habig et al. This content is distributed under the terms of the Creative Commons Attribution 4.0 International license.

**TABLE 2:** Absolute frequencies of novel SNPs and small indels for the aneuploid strains not present in the IPO323 progenitor strain

Strain	Chromosomal status	No. of novel SNPs/indels (∑ = 51)	No. of novel SNPs/indels in coding sequences (∑ = 9)
Zt260	ΔChr14	4	0
Zt295	ΔChr14	6	3
Zt291	ΔChr15	3	2
Zt248	ΔChr15	2	1
Zt251	ΔChr16	2	0
Zt270	ΔChr16	1	0
Zt271	ΔChr17	7	1
Zt299	ΔChr17	8	0
Zt262	ΔChr18	6	0
Zt272	ΔChr18	1	1
Zt290	ΔChr18	1	0
Zt278	ΔChr19	2	0
Zt266	ΔChr20	0	0
Zt279	ΔChr20	2	0
Zt267	ΔChr21	4	0
Zt269	ΔChr21	2	1

### *In vitro* phenotypic variation does not correlate with loss of accessory chromosomes.

We next asked if the loss of accessory chromosomes conferred a fitness effect in the *Z. tritici* strains. We first compared the morphologies of *Z. tritici* colonies grown *in vitro* under various growth conditions (Fig. S5). The strains used for the phenotypic characterizations were derived from independent experiments and therefore represented truly independent chromosomal loss events. The assay allowed us to test the effect of chromosome deletions on growth under conditions of high temperature (28°C), osmotic stress (2 M sorbitol, 1 M NaCl), and oxidative stress (H_2_O_2_) and in the presence of additional cell wall stress agents, including Congo red and calcofluor (27). In general, growth at 28°C increased the extent of colony melanization. However, the observed variation did not correlate with the absence or presence of a particular accessory chromosome (Fig. S5). Likewise, we observed no differences between wild-type and aneuploid strains in colony morphology or growth under the other tested *in vitro* conditions. The only exception was strain Zt266 (with a chromosome 20 deletion [Δchr20]), which showed reduced growth at 28°C and on plates amended with Congo red, calcofluor, sorbitol, and NaCl (Fig. S5).

10.1128/mBio.01919-17.6 FIG S5  *In vitro* phenotypes of IPO323 and aneuploid strains. *In vitro* growth phenotypes were assessed for the chromosome deletion mutants. Cells were grown using different conditions and on different media, including temperature stress and cell wall stress agents. Download FIG S5, PDF file, 0.3 MB. Copyright © 2017 Habig et al. 2017 Habig et al. This content is distributed under the terms of the Creative Commons Attribution 4.0 International license.

### Accessory chromosomes influence the fitness of the aneuploid strains *in planta*.

**FIG 3:**
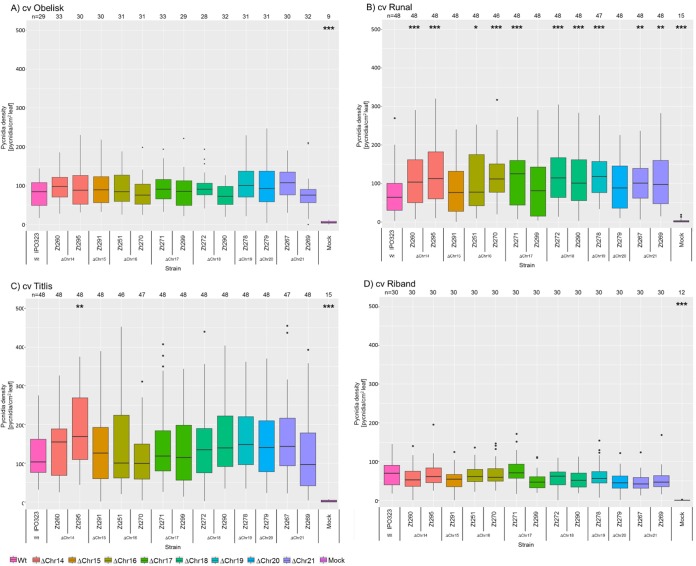
Pycnidia density of the IPO323 progenitor and the aneuploid strains produced at 21 dpi in different wheat cultivars. Box plots are shown for pycnidia density data from wheat cultivars (cv) (A) Obelisk, (B) Runal, (C) Titlis, and (D) Riband. Statistical significance, inferred through an ANOVA and a subsequent *post hoc* Tukey’s HSD test comparing the aneuploid strains to the IPO323 reference, is indicated as follows: *, *P* < 0.05; **, *P* < 0.005; ***, *P* < 0.0005. Data shown are based on results from (A) three, (B and D) four, or (D) two independent experiments.

We next asked to what extent the absence of a particular accessory chromosome affected the fitness of *Z. tritici in planta*. We restricted this analysis to the 13 strains that did not show any large-scale chromosomal aberrations and which we considered to be isogenic. In order to cover a wider range of biotic interactions, four distinct wheat cultivars (Obelisk, Runal, Titlis, and Riband) were included. To quantify disease levels, we measured the density of asexual fruiting bodies at 21 days postinfection (dpi). A minimum of two independent experiments was conducted for each strain under all conditions in a randomized, balanced, and blinded experimental design. The effect of the experimental factors “cultivar,” “strain,” and “experiment” was tested using rank-transformed data in a one-way analysis of variance (ANOVA). The omnibus test showed a significant effect of the factors “cultivar” (*P* = 2 × 10^−16^) and “strain” (*P* = 2 × 10^−16^) and “experiment” (*P* = 2 × 10^−16^) as well as a significant interaction between the factors “strain” and “cultivar” (*P* = 1.1 × 10^−6^). On the basis of these significant interactions, we conclude that the accessory chromosomes affect the phenotype in a cultivar-dependent manner. Further *post hoc* analysis revealed that none of the aneuploid strains varied in the production of pycnidia for the Obelisk cultivar compared to the progenitor IPO323 strain, suggesting that the accessory chromosomes have no effect on the fitness of *Z. tritici* in this wheat cultivar (Fig. 3A). In the Runal cultivar, surprisingly, the wild-type IPO323 strain showed a lower density of pycnidia than most of the tested aneuploid strains (Fig. 3B). These differences were significant for the two strains that had lost chromosome 14 (Zt260 and Zt295), for both strains that had lost chromosome 16 (Zt251 and Zt270), for one of the two tested strains that had lost chromosome 17 (Zt271), for both tested strains that had lost chromosome 18 (Zt272 and Zt290), for the one tested strain that had lost chromosome 19 (Zt278), and for both strains that had lost chromosome 21 (Zt267 and Zt269). For the Titlis cultivar, only strain Zt295, which lacked chromosome 14, showed a significant deviation in pycnidia density from that seen with the progenitor IPO323 strain. For cultivar Riband, none of the tested strains showed a significant difference from the progenitor IPO323 strain. It is interesting that in both cases in which chromosome loss strains, considered to be isogenic, differed in phenotype, we were able to identify an SNP/indel in coding regions of at least one of the strains. Zt271 (Δchr17) shows a nonsynonymous deletion of 20 bp in the coding region of a gene of unknown function (ZT09_chr_1_01551), and ZT295 (Δchr14) contains nonsynonymous SNPs in coding regions of three genes of unknown function. It is possible that these mutations contribute to the observed phenotypic differences between the otherwise isogenic strains (Table S2). Interestingly, the pycnidia density for the aneuploid strains was higher than that for the progenitor IPO323 strain in cultivars Obelisk, Runal, and Titlis, whereas we found a lower pycnidia density (albeit the data were not statistically significant) for the Riband cultivar. Therefore, our results suggest that the independent loss of several of the eight accessory chromosomes confers a gain of fitness in *Z. tritici*, particularly in the Runal wheat cultivar.

### The switch from biotrophy to necrotrophy is influenced by the accessory chromosomes.

**FIG 4:**
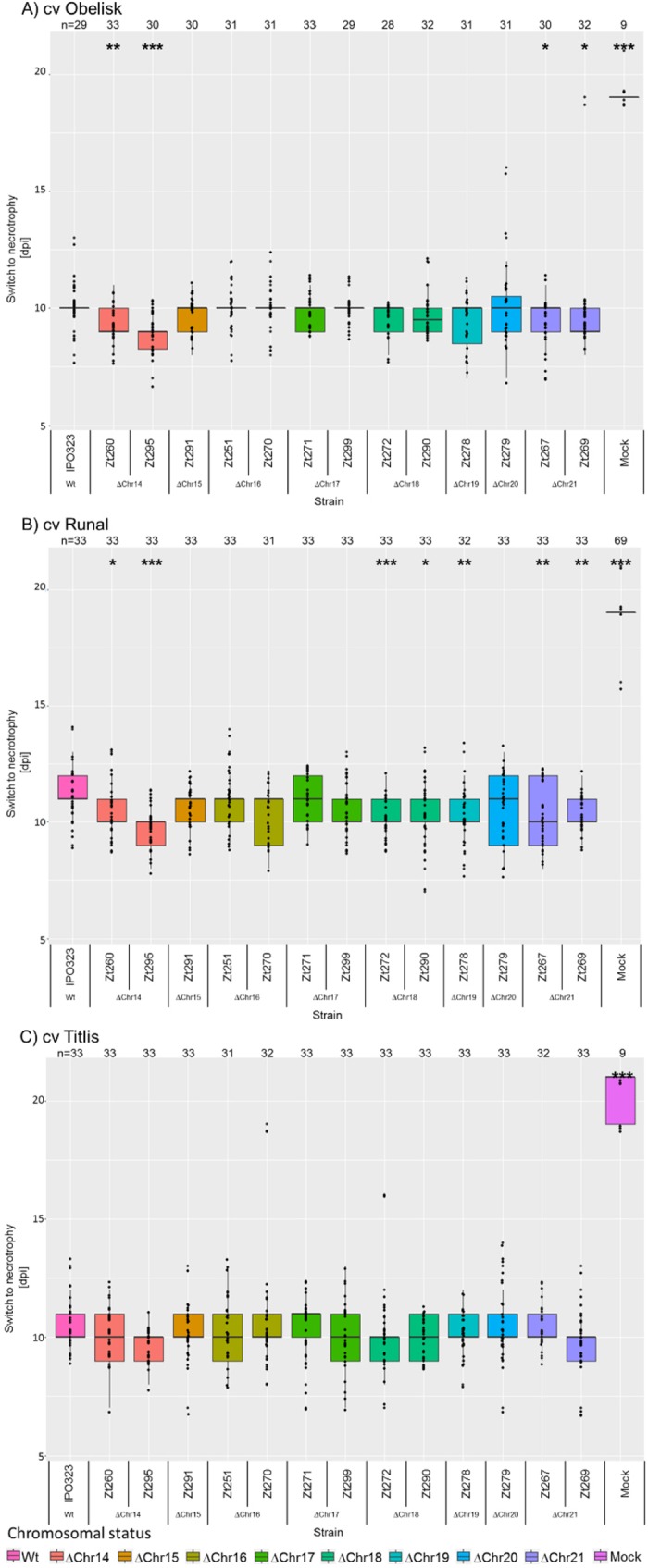
Timing of the switch to necrotrophy of the IPO323 progenitor and the aneuploid strains. (A to C) Boxplots depicting the day postinfection at which the first necrotic symptoms appeared on the leaf surface for cultivars (A) Obelisk, (B) Runal, and (C) Titlis. Data used in the analyses were pooled from results from 3 independent experiments. Statistical significance, inferred through an ANOVA and subsequent *post hoc* Tukey’s HSD test comparing the aneuploid strains to the IPO323 reference, is indicated as follows: *, *P* < 0.05; **, *P* < 0.005; ***, *P* < 0.0005.

The temporal development of disease of *Z. tritici* can vary between isolates and cultivars (M. Habig, unpublished data), and we hypothesized that the temporal development of *Z. tritici in planta* could be influenced by the presence/absence of certain accessory chromosomes. In particular, the timing of the switch from biotrophy to necrotrophy may affect the overall fitness of *Z. tritici* as the switch is crucial for the nutrient availability of the fungus and thus for its ability to produce pycnidia. Therefore, to test if the accessory chromosomes contribute to temporal disease development, we monitored the inoculated leaves daily to compare data corresponding to symptom development for the aneuploid strains on the three wheat cultivars Obelisk, Runal, and Titlis in three independent experiments (Fig. 4). The effects of the experimental factors “cultivar,” “strain,” and “experiment” were tested using rank transformation in a one-way ANOVA. The omnibus test showed a significant effect of the factors “cultivar” (*P* = 2 × 10^−16^) and “strain” (*P* = 2 × 10^−16^) and “experiment” (*P* = 2 × 10^−16^). However, the interaction between the factors “strain” and “cultivar” was statistically nonsignificant. Therefore, the effect of the accessory chromosomes on the timing of the switch to necrotrophy is independent of the cultivar. *Post hoc* analysis showed that both independent strains lacking chromosome 14 (Zt260 and Zt295) showed an earlier onset of necrosis than the IPO323 progenitor strain on the Runal wheat cultivar. Similarly, the two strains without chromosome 18 (Zt272 and Zt290) varied significantly from the progenitor IPO323 strain. Strain Zt278 lacking chromosome 19 and the two strains without chromosome 21 (Zt267 and Zt269) caused symptoms significantly earlier than IPO323. On the Obelisk cultivar, the strains lacking chromosomes 14 and 21 showed a significantly earlier onset of necrosis. However, as shown above, the temporal variation of disease development on Obelisk did not translate into differences in the total disease symptoms measured at 21 dpi (Fig. 3A). On the Titlis cultivar, no aneuploid strain showed significant differences from the IPO323 strain results. From our *in planta* assay results, we thereby conclude that the temporal development of fungal infection is influenced by the complement of accessory chromosomes but that this effect is independent of the host cultivar.

### The timing of the switch to necrotrophy impacts the level of pycnidia formation in a wheat cultivar-dependent manner.

We next addressed the issue of whether the timing of the switch to necrotrophy influences the number of pycnidia produced by the fungus. An earlier switch to necrotrophy should be correlated with a higher number of pycnidia as the fungus would have a longer phase during which to take up nutrients released from dead plant tissue. We also considered the possibility that the timing of the switch could be determined by host genotype. To test the influence of the host genotype, we pooled data for all fungal strains on the three wheat cultivars Obelisk, Runal, and Titlis and correlated the time of appearance of necrotic symptoms on individual leaves with the corresponding pycnidia density obtained for the corresponding leaf at 21 dpi. The balanced experimental design, in which all three cultivars were tested in same three experiments with the same fungal strains, allowed us to pool data for all fungal strains and to compare the effects of the cultivars without the confounding effect of the fungal strains. However, a lack of statistical power prevented performance of the analysis with a nonpooled data set, i.e., on the level of individual accessory chromosomes.

Both “cultivar” and “switch to necrotrophy” were identified as significant factors influencing pycnidia density in a one-way ANOVA performed on rank-transformed data (28) (*P* < 2 × 10^−16^ and *P* < 2 × 10^−16^, respectively). To test whether the cultivars influenced the timing of the switch to necrotrophy, the interaction of these two factors was also tested. The interaction proved to be highly statistically significant (*P* = 3.5 × 10^−6^), indicating that the three cultivars influence the shape of the correlation between the switch to necrotrophy and the pycnidia density. A *post hoc* pairwise comparison revealed that the shapes of the curves were significantly different between the Titlis cultivar and Obelisk (*P* = 7.0 × 10^−6^), and we therefore conclude that the timing of the switch impacts the level of pycnidia formation in a cultivar-dependent manner.

**FIG 5:**
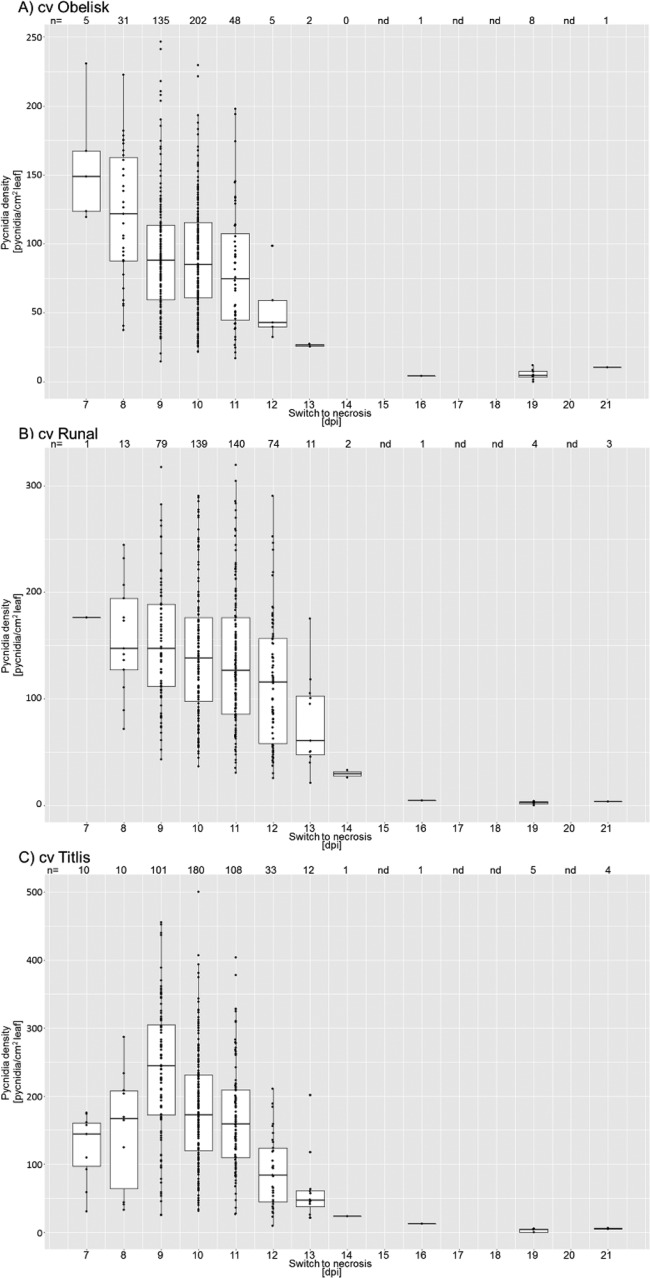
The timing of switch to necrotrophy influences the number of pycnidia produced. (A to C) Boxplots of the pycnidia density on leaves at 21 dpi grouped according to the day (postinfection) when the first necrotic symptoms appeared on the respective leaf for cultivars (A) Obelisk, (B) Runal, and (C) Titlis. Data for all tested *Z. tritici* strains were pooled for each of the cultivars. The shapes of the curves as tested by the interaction of the factor cultivar and the switch to necrotrophy proved significantly different for the pairwise comparisons of Obelisk and Titlis (*P* = 7.0 × 10^−6^).

In detail, the number of pycnidia seen with the Titlis wheat cultivar was lower on plants where the first necrosis symptoms developed at 7 and 8 dpi than on those plants that developed the first symptoms at 9 dpi (Fig. 5C). In contrast, for the Obelisk wheat cultivar, the pycnidia density was higher on leaves on which the first symptoms appeared at 7 and 8 dpi than on leaves on which the first symptoms appeared at 9 dpi (Fig. 5A). For the Runal wheat cultivar, the pycnidia density showed little variation from 7 to 9 dpi (Fig. 5B). For all three wheat cultivars, however, the density of pycnidia decreased when the first symptoms developed later than 9 dpi. An early switch from biotrophic growth to necrotrophic growth thereby correlated with less pycnidia production and lower fitness on the Titlis wheat cultivar, while earlier disease development correlated with increased pycnidia production and higher fitness of the pathogen on Obelisk. Thus, the host genotype can impact the fitness advantage of different *Z. tritici* strains based on their ability to develop disease early or late after infection.

## DISCUSSION

The genome of *Z. tritici* can contain a large number of accessory chromosomes; however, a functional relevance of these chromosomes has so far not been directly shown. On the basis of the forward genetics approach applied here, we could demonstrate, for the first time, that the accessory chromosomes affect the phenotype of *Z. tritici in planta*. Most notably, the effects of the accessory chromosomes are dependent on host genotype. The deletion of accessory chromosomes 14, 16, 18, 19, and 21 led to an increase in the pycnidia density in the Runal wheat cultivar, whereas no effect was detected in wheat cultivars Obelisk, Titlis, and Riband. The results were consistent for four of five pairs of independent chromosome loss mutants, i.e., pairs of strains that had lost the same accessory chromosome. On Runal, however, one chromosome loss strain (Δchr17) showed significantly increased production of pycnidia, while the other Δchr17 strain did not. Similarly, on Titlis, only one of the two Δchr14 strains showed increased production of pycnidia. Our comparison of full-genome sequences revealed that these otherwise isogenic strains deviate with additional SNP/indels in coding regions. We hypothesize that these extra mutations may explain the observed differences between the two sets of chromosome loss strains. The switch from biotrophy to necrotrophy was also affected by the accessory chromosomes—those effects, however, proved to be similar for all three tested cultivars and therefore not dependent on the host genotype.

The finding of an increased pycnidia density in the Runal wheat cultivar upon deletion of accessory chromosomes 14, 16, 18, 19, and 21 suggests that these chromosomes reduce the fitness of *Z. tritici* in a cultivar-dependent manner. One explanation could be that chromosomes 14, 16, 18, 19, and 21 express factors that are recognized by the Runal cultivar and induce partial immunity either via pathogen-associated-molecular-pattern-triggered immunity (PTI) or via effector-triggered immunity (ETI). *Z. tritici* was previously shown to induce PTI in wheat upon recognition of chitin in the fungal cell wall. *Z. tritici* secretes Mg3LysM and Mg1LysM, the two LysM effectors that bind chitin with high affinity to prevent PTI (2, 29). However, accessory chromosomes of *Z. tritici* may encode avirulence genes or other secreted molecules that specifically trigger defense reactions in some wheat cultivars.

Interestingly, the observed effects of the accessory chromosomes on the phenotype are quantitative; i.e., they are small but significant. Our findings of quantitative effects of the accessory chromosomes are in agreement with a recent study of *Z. tritici* based on quantitative trait locus (QTL) mapping of virulence-associated loci. In this study, similar small effects of the accessory chromosome 18 on melanization and pycnidia density were demonstrated in the Titlis cultivar and of chromosome 21 on pycnidia size in the Runal cultivar (25). These small effects contrast with the scenario in *N. haematococca* and *F. oxysporum* (f. sp. *lycopersici*), where accessory genetic elements are the determining factors for genotype-specific pathogenicity (12–14). However, even though the effects of accessory chromosomes in *Z. tritici* are small, our results show that they can contribute to fitness differences on different hosts. Surprisingly, the accessory chromosomes seem to have a negative fitness effect that should lead to a complete loss of the accessory chromosomes unless they also provide a fitness advantage under other conditions or at other stages of the life cycle.

The localization of pathogenicity-related genes on the accessory chromosomes may be a route by which to rapidly generate phenotypic variation on which natural selection can act. The localization of pathogenicity-related genes on accessory chromosomes may reflect past adaptations to more-diverse host populations in natural ecosystems. In this context, it is interesting that the accessory chromosomes of *Z. tritici* show some homology to accessory chromosomes of the sister species *Z. pseudotritici* and *Z. ardabiliae* infecting wild grasses (22, 30, 31). The finding of conserved accessory chromosome fragments among different *Zymoseptoria* species shows that the accessory chromosomes represent an ancient trait that predates the diversification of species in the *Zymoseptoria* genus. *Z. pseudotritici* and *Z. ardabiliae* infect wild grass species and have a wider host range than *Z. tritici* (31). It is conceivable that the accessory chromosomes not only determine wheat cultivar specificity in *Z. tritici* but also play a role in determining the host range of *Z. pseudotritici* and *Z. ardabiliae*.

An intriguing finding of this study is that the accessory chromosomes affect the timing of the switch from biotrophy to necrotrophy. In addition, we find pronounced differences between the three cultivars with respect to the correlation between the onset of necrosis and the number of pycnidia produced. An early switch to necrotrophy is correlated with a higher density of pycnidia in the Obelisk cultivar but with a lower pycnidia density in Titlis. The early onset of necrosis in the Titlis wheat cultivar might be associated with an earlier recognition of the fungus by the plant, resulting in plant defenses that in turn reduce the number of pycnidia that the fungus can produce. The differences in the correlations between the first symptoms of necrosis and the density of pycnidia for the three cultivars indicate that plant factors influence the temporal development of the disease symptoms in combination with an effect of accessory chromosome complements.

Although the accessory chromosomes are relatively gene poor (1), comprise mainly heterochromatic DNA (16), and in general are transcribed at a considerably lower level than the core chromosomes, several genes are strongly upregulated during infection (3, 15). Such differential gene expression levels support the idea of the functional importance of genes on the accessory chromosomes as also suggested by our study results. A total of nine effector candidates can be identified on the accessory chromosomes by computational predictions from secretome data (32). Among the effector candidates, one locates on chromosome 14, two on chromosome 16, two on chromosome 18, and one on chromosome 21. These and the differentially expressed genes from chromosomes 14 and 19 identified in previous studies (3, 15) are promising candidates for further genetic and functional analyses.

In conclusion, we demonstrate here for the first time that the large complement of accessory chromosomes in the wheat pathogen *Z. tritici* plays a role in fitness during host infection. The effects were found for five of the eight accessory chromosomes and affected two fitness-related traits. Surprisingly, we identified significant fitness costs of the accessory chromosomes only, suggesting that other factors or selective traits act to maintain the chromosomes in the pathogen populations. One of the fitness traits found to be affected in this study, pycnidia density, is affected in a wheat cultivar-dependent manner, strongly suggesting an interaction between host genes and genes harbored by the accessory chromosomes of *Z. tritici*.

## MATERIALS AND METHODS

### Fungal and plant material.

The Dutch IPO323 isolate was kindly provided by Gert Kema (Wageningen, the Netherlands) and is available from the Centre of the Royal Netherlands Academy of Arts and Sciences (Utrecht, the Netherlands) under accession number CBS 115943. Strains were maintained in either liquid yeast-malt-sucrose (YMS) broth (4 g/liter yeast extract, 4 g/liter malt extract, 4 g/liter sucrose) at 18°C on an orbital shaker or on solid YMS (with 20 g/liter agar added) at 18°C. The *Triticum aestivum* cultivar Obelisk was obtained from Wiersum Plantbreeding BV (Winschoten, the Netherlands). Wheat cultivars Runal and Titlis were obtained from DSP AG (Delley, Switzerland). Wheat cultivar Riband was kindly provided by Jason Rudd (Rothamsted Research, Harpenden, United Kingdom).

### Induction of aneuploidy.

IPO323 (local identifier [ID] Zt244) maintained on YMS solid medium was inoculated at 200 cells/ml into YMS liquid media with 0 to 3 µg/ml carbendazim (Sigma-Aldrich, Munich, Germany) at 18°C with shaking at 200 rpm. At 7 days postinoculation, the cell suspension was transferred into a new test tube and the incubation continued for another 7 days. At 7 and 14 days postinoculation, cells were streaked onto YMS solid plates and incubated for 7 days at 18°C. If available, eight colonies from each treatment were karyotyped by PCR using multiplexed primers targeting subtelomeric regions of the accessory chromosomes (see below). If necessary, the loss of a specific accessory chromosome was confirmed by additional PCR along the chromosome.

### PCR karyotyping.

We initially karyotyped carbendazim-treated colonies using a PCR approach. DNA of a single colony was isolated by resuspending cells in 50 µl 25 mM NaOH followed by 10 min at 98°C and a neutralization step performed by adding 150 µl 13.3 mM TrisHCl (pH 5.5). Two multiplexed PCRs were developed using eight primer pairs. Each of the eight primer pairs was designed to bind in the subtelomeric region of chromosome 14, 15, 16, 17, 18, 19, 20, and 21, respectively. PCR was performed using Phusion DNA polymerase (New England Biolabs, Frankfurt, Germany) (0.04 units/µl) with 0.2 mM deoxynucleoside triphosphates (dNTPs), 3% dimethyl sulfoxide (DMSO), and a 1.6 µM concentration of each primer in a total volume of 7 or 10 µl. If a colony showed no amplicon for one accessory chromosome, the colony was propagated and DNA was isolated using a standard phenol-chloroform protocol (33). To confirm the loss of an accessory chromosome, at least three independent PCRs for each accessory chromosome were performed. To verify the performance of each of these PCRs, a primer pair amplifying a section of the housekeeping GAPDH (glyceraldehyde-3-phosphate dehydrogenase) gene was included as an internal control. An accessory chromosome was considered lost only if all PCRs specific for the accessory chromosomes confirmed the absence of the accessory chromosome while showing amplification of the GAPDH locus. See Table S3 in the supplemental material for a list of all primers used in this study.

10.1128/mBio.01919-17.9 TABLE S3  List of all primers used in this study. Download TABLE S3, XLSX file, 0.01 MB. Copyright © 2017 Habig et al. 2017 Habig et al. This content is distributed under the terms of the Creative Commons Attribution 4.0 International license.

### Pulsed-field gel electrophoresis (PFGE).

Verification of the karyotype was conducted using a pulsed-field gel electrophoresis system (DRII; Bio-Rad, Munich, Germany). Plugs containing intact chromosomes were produced from a *Z. tritici* cell suspension by centrifugation (3,500 × *g*, 10 min) and resuspension of the cell pellet in 1 ml double-distilled water (ddH_2_O). Whole cells were embedded in 1 ml 2.2% Low-Range Ultra Agarose (Bio-Rad, Munich, Germany)–0.5× Tris-borate-EDTA (TBE) buffer and incubated twice for 24 h each time at 55°C in 5 ml lysis buffer (1.5 mg/ml proteinase K, 1% SDS, 0.45 M EDTA, pH 8.0). Small chromosomes were separated in 1.2% pulsed-field agarose (Bio-Rad, Munich, Germany)–0.5× TBE buffer and were processed for 48 h at 14°C using an angle of 120°, 5 V/cm, and a switching time of 50 to 150 s. Midsize chromosomes were separated in 1% pulsed-field agarose–1× TBE buffer and were processed for 72 h at 14°C using an angle of 106°, 3 V/cm, and a switching time of 250 to 1,000 s. Large chromosomes were separated in 0.8% pulsed-field agarose–1× TRIS-acetate-EDTA (TAE) buffer and were processed for 92 h at 13°C using an angle of 106°, 2 V/cm, and a switching time of 1,000 to 2,000 s. Gels were stained for 30 min in a 0.5 µg/ml ethidium bromide solution followed by 10 min of destaining in H_2_O.

### Genome sequencing.

For sequencing, a minimum of 2 µg DNA was isolated using a previously described phenol-chloroform extraction protocol (33). Sequencing was performed using an Illumina HiSeq 3000 system and included only two rounds of PCR-based amplification in order to minimize the number of PCR artifacts. Paired-end reads of 150 bp were sequenced for each strain. Sequencing was performed at the Max Planck-Genome-centre Cologne, Cologne, Germany. The reads determined in this work were deposited in the Sequence Read Archive (see below).

### Data handling.

Specific parameters for Illumina read processing are summarized in Text S1 in the supplemental material. In short, the program Trimmomatic (34) was used for quality filtering of the read data (using the following parameter settings: headcrop, 2; crop, 149; leading, 3; trailing, 3; slidingwindow, 4:15; Minlen, 50). Reads were mapped to the reference genome of IPO323 using bowtie2 (35). Reads mapping two or more times were removed using Picard (http://broadinstitute.github.io/picard). Major rearrangements and whole-chromosome deletions were assessed by inspection of the genome-wide read coverage. SNPs and indel mutations were identified using SAMtools software (36) with the function mpileup, applying a minimum base quality threshold of 20 and a ploidy level of 1 for all chromosomes. SNPs and indels with a quality value of <20 and a read depth threshold (DP) value of <10 were discarded. According to the haploid genome structure of *Z. tritici*, we further discarded sites with an allele frequency of <0.8%.

10.1128/mBio.01919-17.1 TEXT S1  Protocol of read processing, reference assembly, and SNP calling. Download TEXT S1, PDF file, 0.3 MB. Copyright © 2017 Habig et al. 2017 Habig et al. This content is distributed under the terms of the Creative Commons Attribution 4.0 International license.

### Determination of the *Z. tritici* phenotypes *in vitro* and *in planta.*

The *Z. tritici* strains were grown on YMS solid medium for 5 days at 18°C before the cells were scraped from the plate surface. The cell number was adjusted to 10^7^ cells/ml in ddH_2_O, and the reaction mixture was serially diluted to 10^3^ cells/ml. A 3-μl volume of each cell dilution was transferred onto YMS agar that included the test compounds, and the reaction mixture was incubated for 7 days at 18°C or 28°C. For the tests using high osmotic stresses, 1 M NaCl and 2 M sorbitol (obtained from Carl Roth GmbH, Karlsruhe, Germany) were added to the YMS solid medium. For the tests using cell wall stresses, 500 µg/ml Congo red and 200 µg/ml calcofluor (obtained from Sigma-Aldrich Chemie GmbH, Munich, Germany) were added to the YMS solid medium. To test the effect of reactive oxygen species, 2 mM H_2_O_2_ (obtained from Carl Roth GmbH, Karlsruhe, Germany) was added to the YMS solid medium.

For the *in planta* phenotypic assays, we germinated seeds of the four wheat cultivars Obelisk, Runal, Titlis, and Riband on wet sterile Whatman paper for 4 days before potting was performed using Fruhstorfer Topferde soil (Hermann Meyer GmbH, Rellingen, Germany). Wheat seedlings were further grown for 7 days before inoculation. *Z. tritici* strains were grown on YMS solid medium for 5 days at 18°C before the cells were scraped from the plate surface. The cell number was adjusted to 10^7^ cells/ml in H_2_O with 0.1% Tween 20. The cell suspension was brushed onto approximately 5 cm on the abaxial and adaxial sides of the second leaf of each seedling. Inoculated plants were placed in sealed bags containing water for 48 h to facilitate infection through stomata. Plants were grown under constant conditions with a day/night cycle of 16 h of light (~200 µmol/m^2^/s) and 8 h of darkness in growth chambers at 20°C. Plants were grown for 21 days postinoculation at 90% relative humidity (RH). For determining the onset of necrosis, the leaves were visually inspected for the appearance of symptoms at 7, 8, 9, 10, 11, 12, 13, 14, 16, 19, and 21 dpi within three experiments using the wheat cultivars Obelisk, Runal, and Titlis. The day of the first appearance of symptoms was recorded for each plant. Upon completion of the experiment, the infected leaves were cut and taped to sheets of paper and pressed for 5 days at 4°C before being scanned at a resolution of 2,400 dpi using a flatbed scanner (HP Photosmart C4580; HP, Böblingen, Germany). Scanned images were analyzed using automated image analysis and ImageJ (37) and a method adapted from reference 38. The readout (number of pycnidia per square centimeter of leaf surface) was used for all subsequent analyses. See Table S4 for a summary of all *in planta* results.

10.1128/mBio.01919-17.10 TABLE S4  Summary of *in planta* results. Download TABLE S4, XLSX file, 0.3 MB. Copyright © 2017 Habig et al. 2017 Habig et al. This content is distributed under the terms of the Creative Commons Attribution 4.0 International license.

Statistical analyses were conducted in R (version r3.4.1) (39) using the suite RStudio (version 1.0.143) (40). Data inspection showed a nonnormal distribution for all data sets, including the measured pycnidia density (number of pycnidia per square centimeter) and the data for the switch to necrotrophy and thus also the relationship between the two. Therefore, we performed an omnibus analysis of variance using rank transformation of the data (28). For the three data sets, we performed calculations using the following models: (i) pycnidia density ~ strain * cultivar * experiment; (ii) switch to necrotrophy ~ strain * cultivar * experiment; and (iii) pycnidia density ~ switch to necrotrophy * strain * cultivar * experiment. In all cases, *post hoc* tests were performed using Tukey’s honestly significant difference (HSD) test (41).

### Accession number(s)*.*

The reads determined in this work were deposited in the Sequence Read Archive with BioProject ID PRJNA371572.
