# Supplementary material for: Forward Genetics Approach Reveals Host Genotype-Dependent Importance of Accessory Chromosomes in the Fungal Wheat Pathogen Zymoseptoria tritici
Source: mBio. 2017 Nov 28;8(6):e01919-17. doi: 10.1128/mBio.01919-17 (PMC5705923; doi:10.1128/mBio.01919-17)
Supplement: FIG S1 [file mbo006173611sf1.pdf]

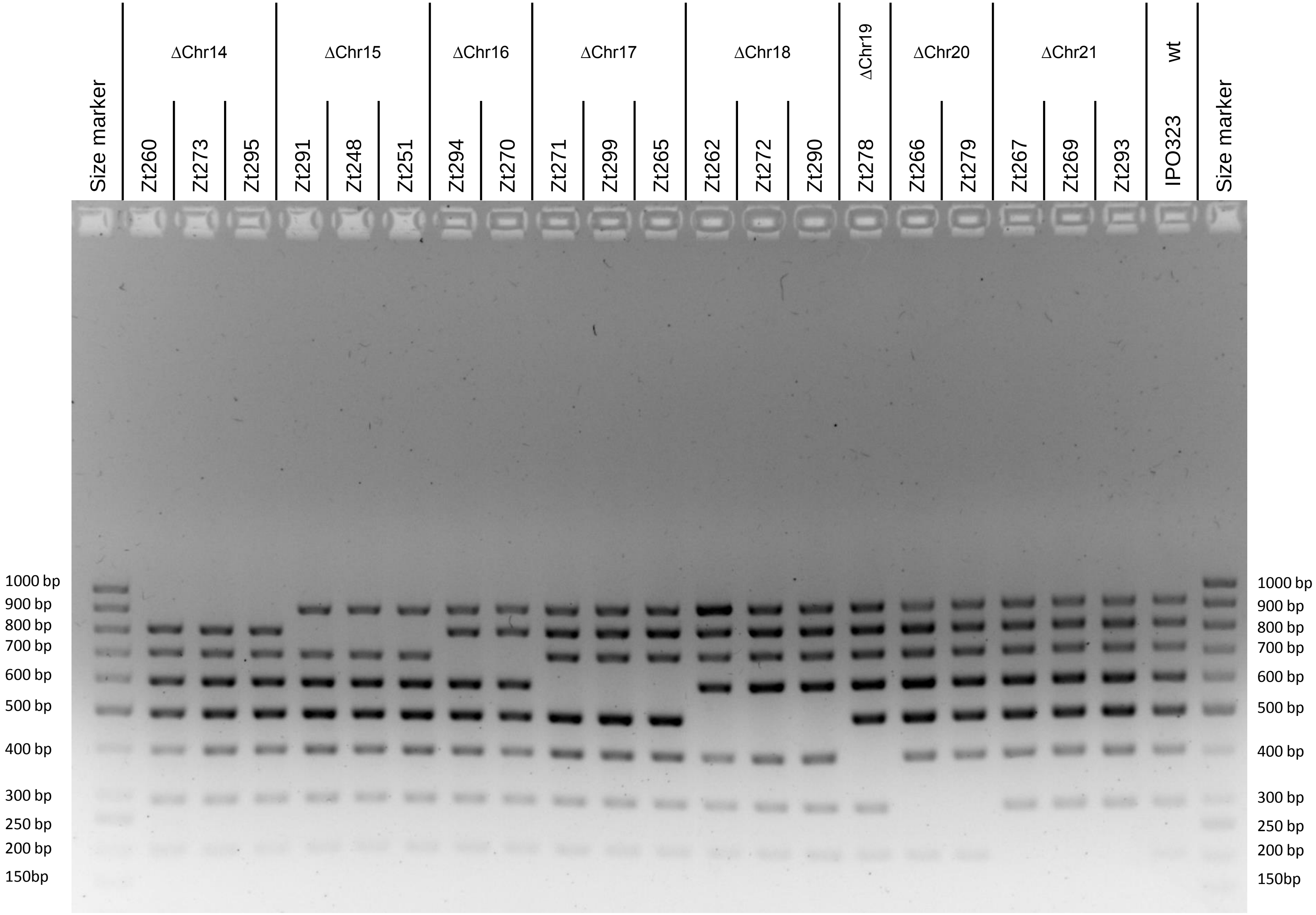

Chromosome	Region	Primer-No	expected product size [bp]
Chr 14	Subtelomeric R	2086 x 2087	900
Chr 15	Subtelomeric R	2090 x 2091	800
Chr 16	Subtelomeric R	2094 x 2095	700
Chr 17	Subtelomeric R	2098 x 2099	600
Chr 18	Subtelomeric R	2102 x 2103	500
Chr 19	Subtelomeric R	2106 x 2017	400
Chr 20	Subtelomeric R	2110 x 2111	300
Chr 21	Subtelomeric R	2114 x 2115	200

FIG S1 A Gel electrophoresis of multiplexed PCR targeting the subtelomeric region of chromosome 14-21.

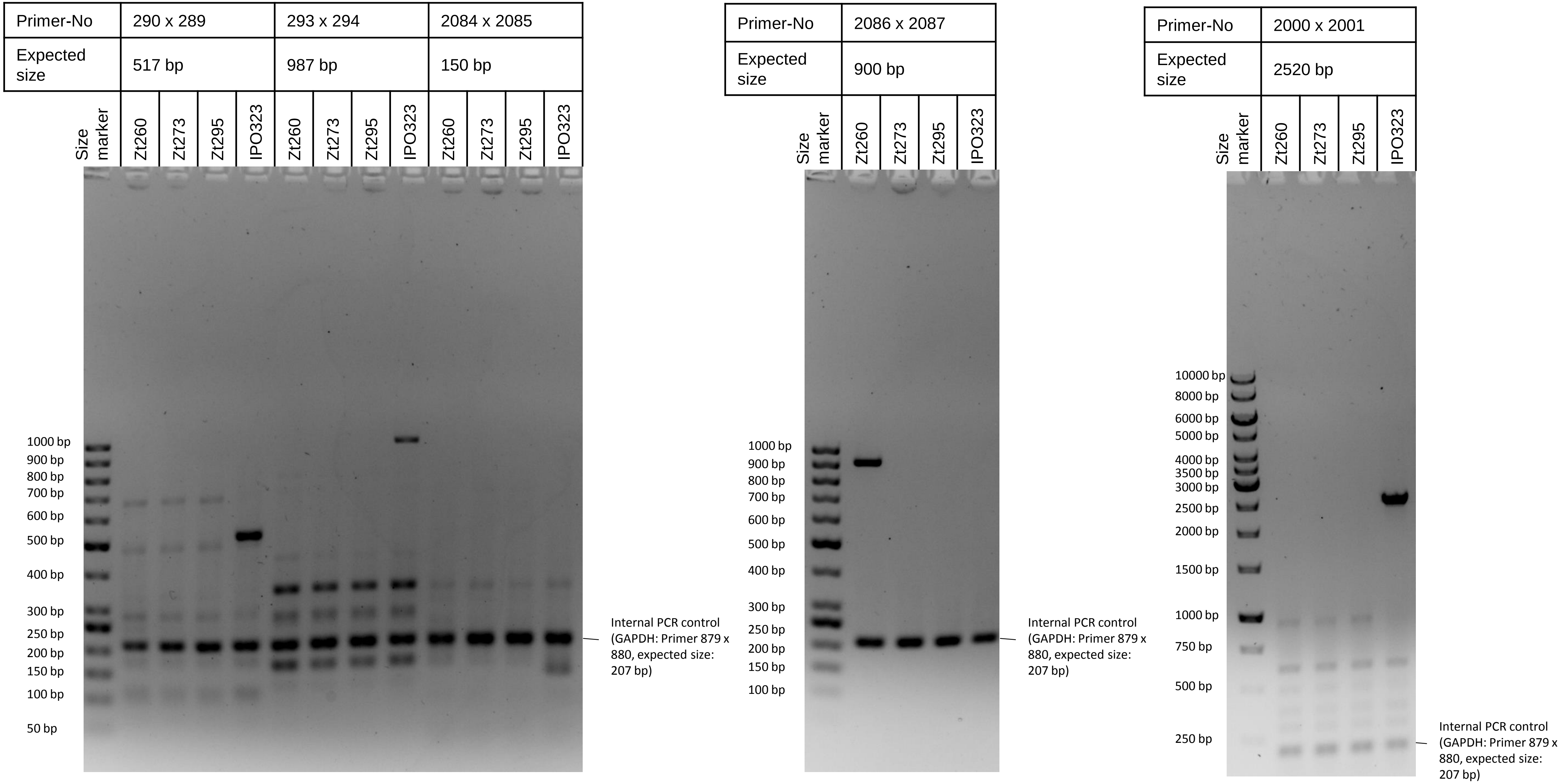

FIG S1 B Gel electrophoresis of PCR along the chromosome 14 for strains that had lost chromosome 14.

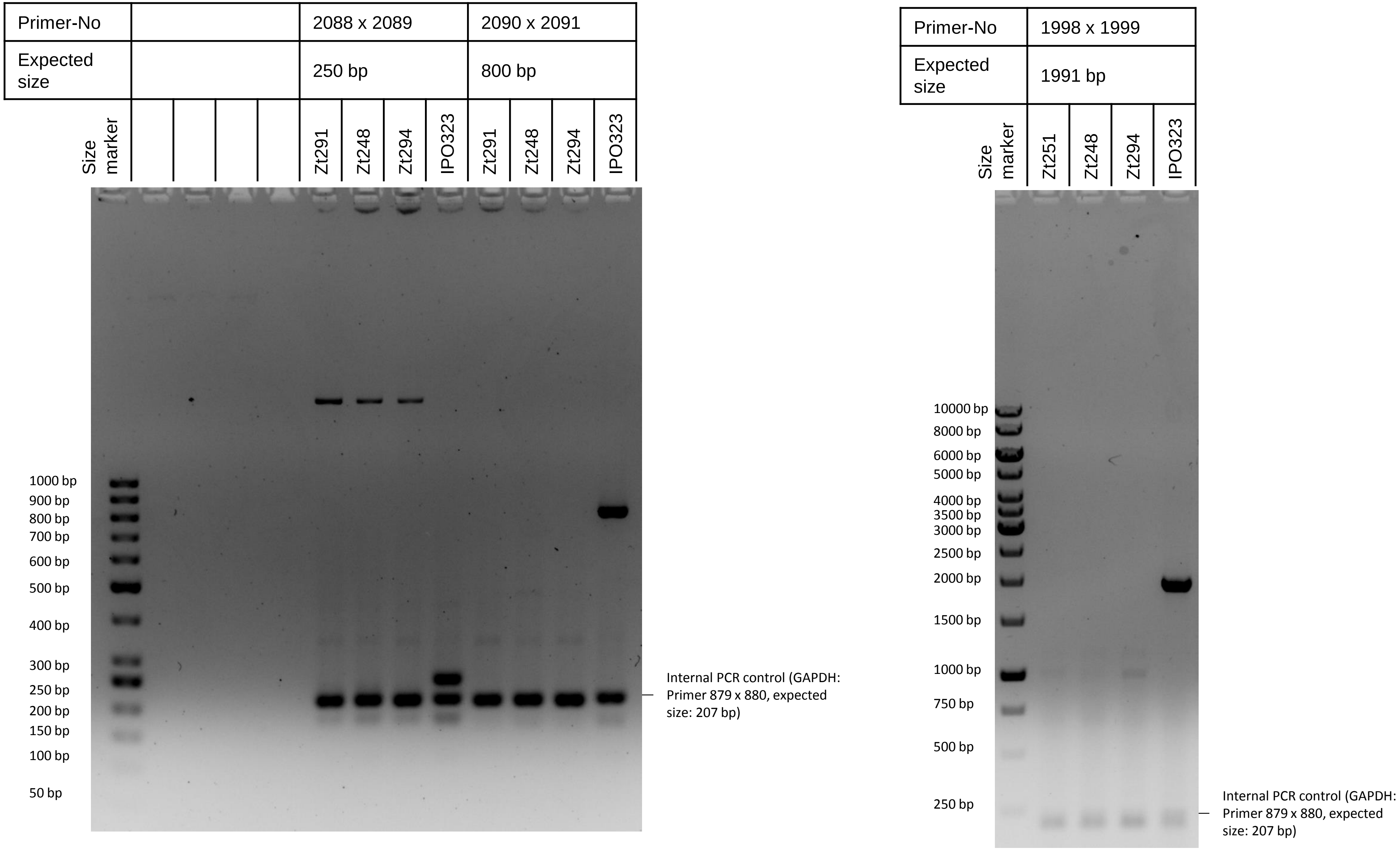

FIG S1 C Gel electrophoresis of PCR along the chromosome 15 for strains that had lost chromosome 15.

Primer-No	1010 x 1009	2092 x 2093	2094 x 2095
Expected size	104 bp	350 bp	700 bp

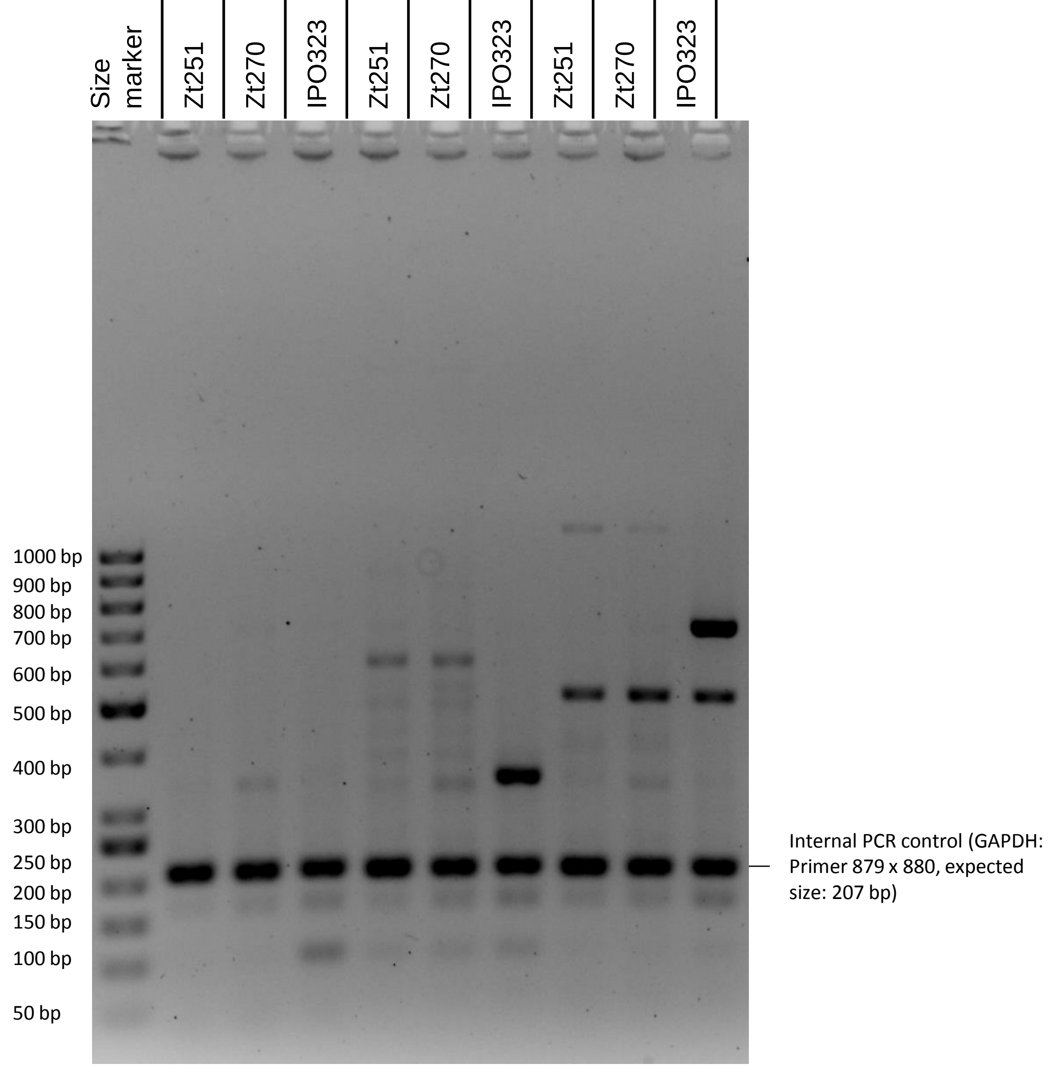

Primer-No	1996 x 1997
Expected size	1501 bp

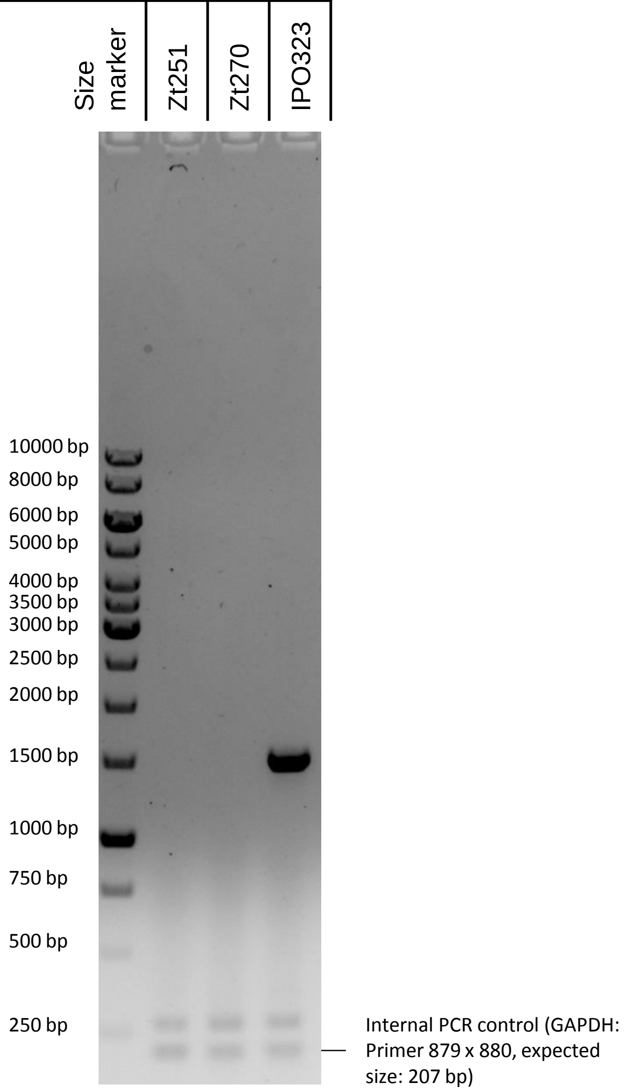

FIG S1 D Gel electrophoresis of PCR along the chromosome 16 for strains that had lost chromosome 16.

Primer-No	1263 x 1264				1995 x 1994				2096 x 2097				2094 x 2095			
Expected size	139 bp				1190 bp				450 bp				600 bp			
Size marker	Zt271	Zt299	Zt265	IPO323	Zt271	Zt299	Zt265	IPO323	Zt271	Zt299	Zt265	IPO323	Zt271	Zt299	Zt265	IPO323

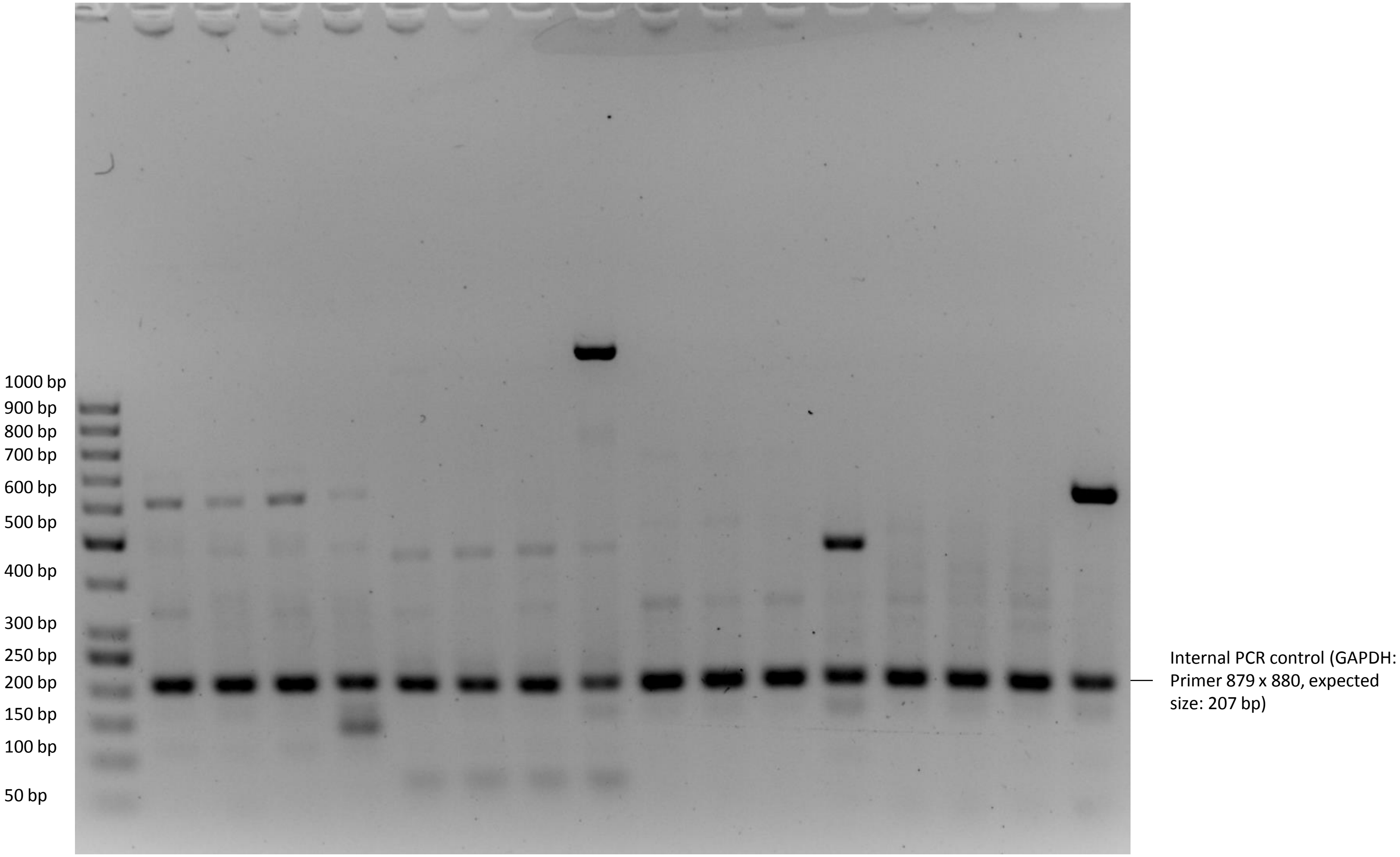

FIG S1 E Gel electrophoresis of PCR along the chromosome 17 for strains that had lost chromosome 17.

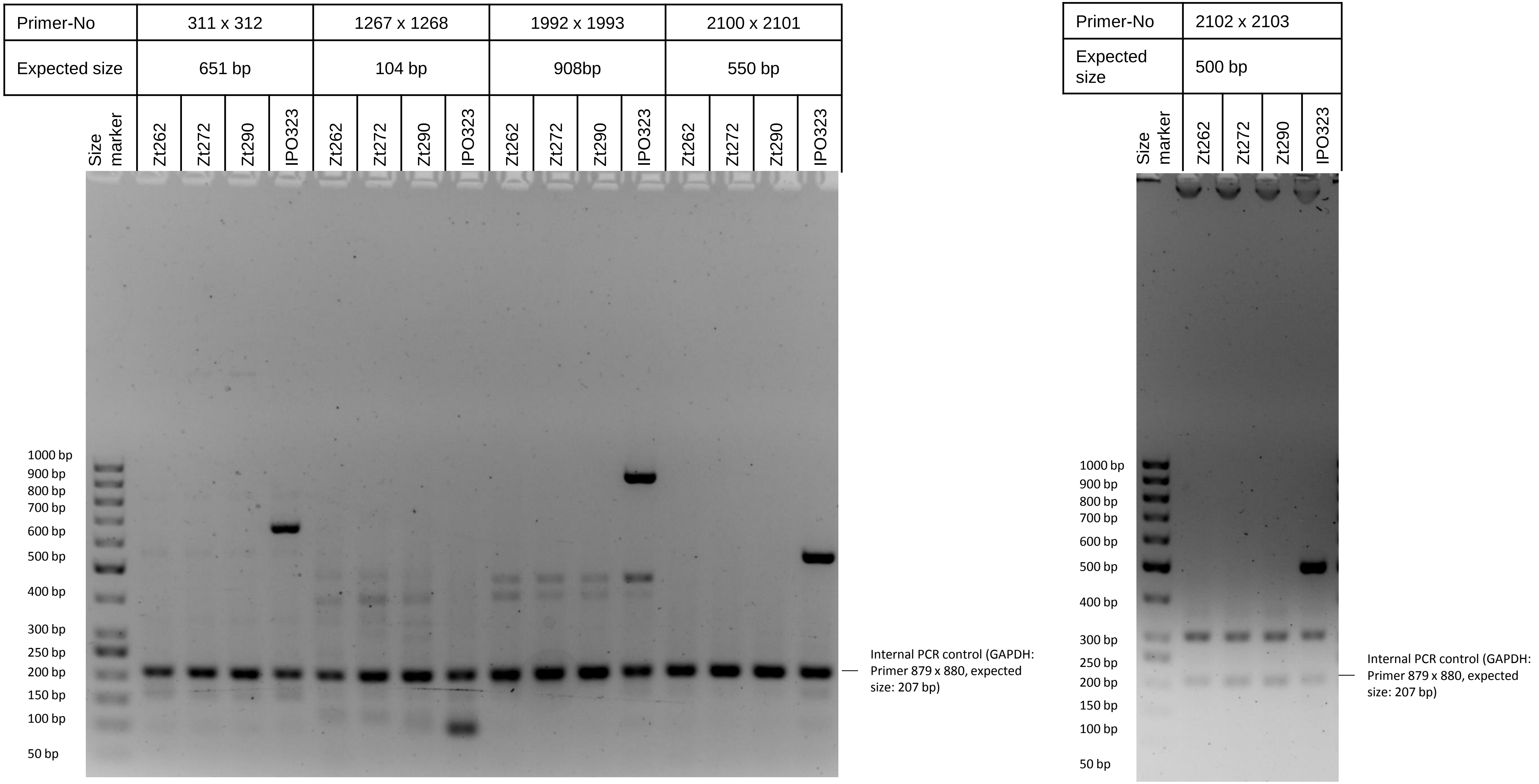

FIG S1 F Gel electrophoresis of PCR along the chromosome 18 for strains that had lost chromosome 18.

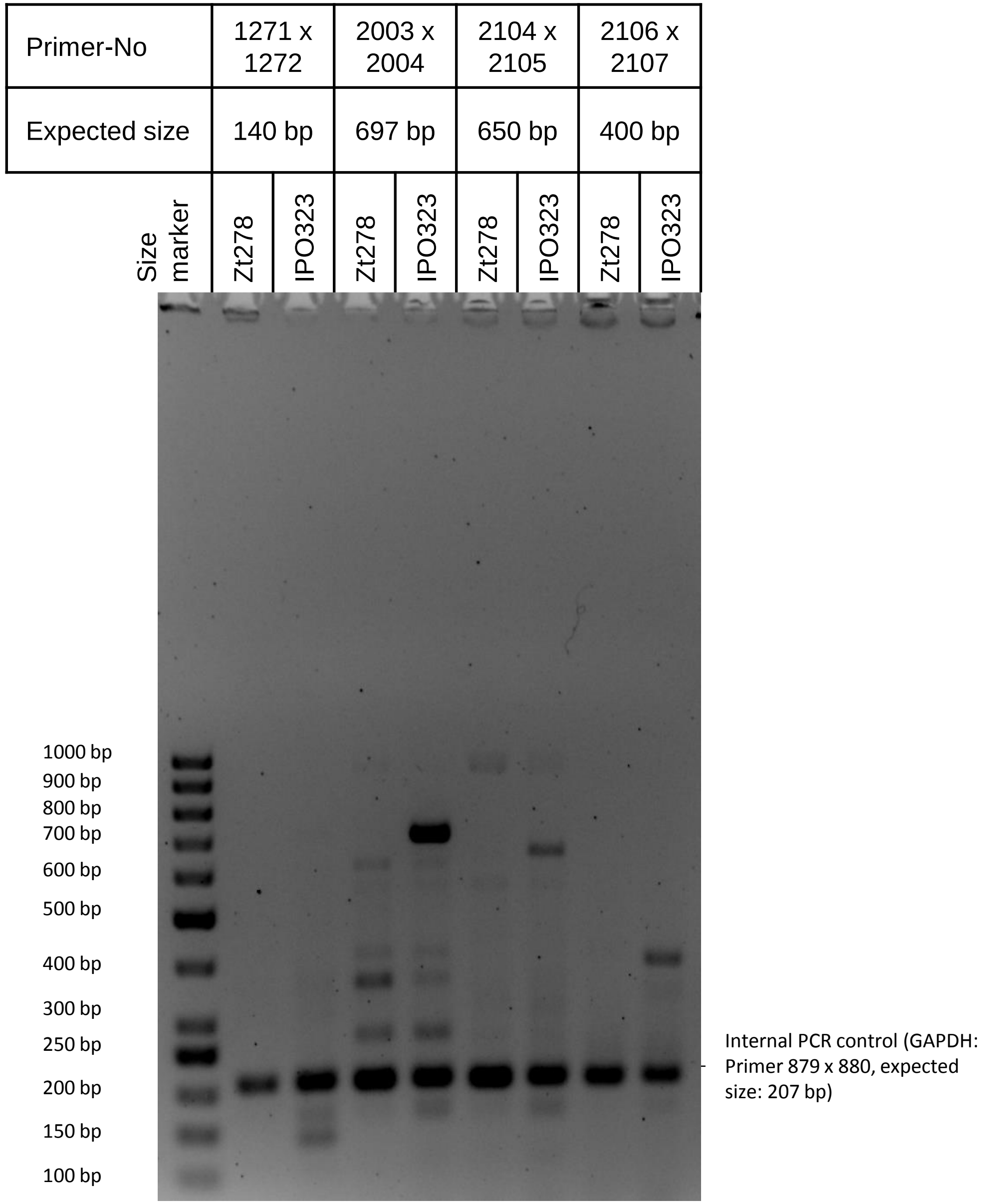

FIG S1 G Gel electrophoresis of PCR along the chromosome 19 for strains that had lost chromosome 19.

Primer-No	1276 x 1275			2110 x 2111			2108*2109		
Expected size	142 bp			300 bp			750 bp		
Size marker	Zt266	Zt279	IPO323	Zt266	Zt279	IPO323	Zt266	Zt279	IPO323

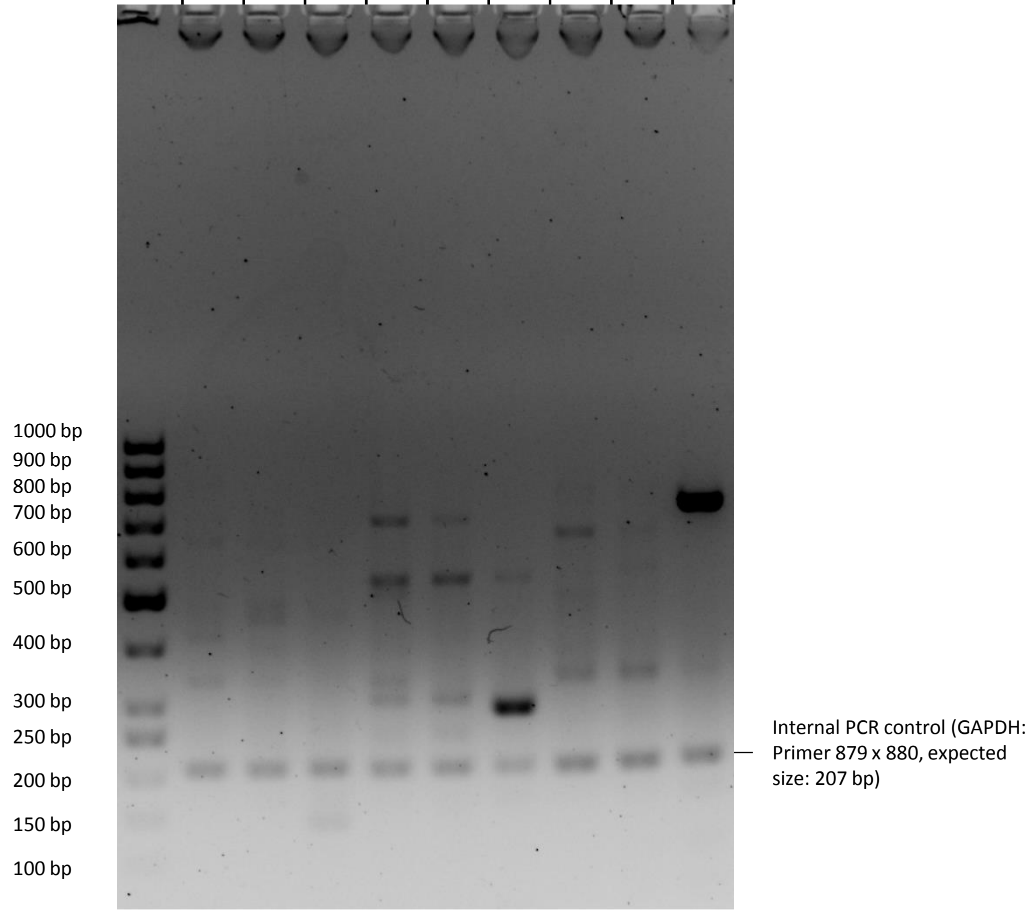

FIG S1 H Gel electrophoresis of PCR along the chromosome 20 for strains that had lost chromosome 20.

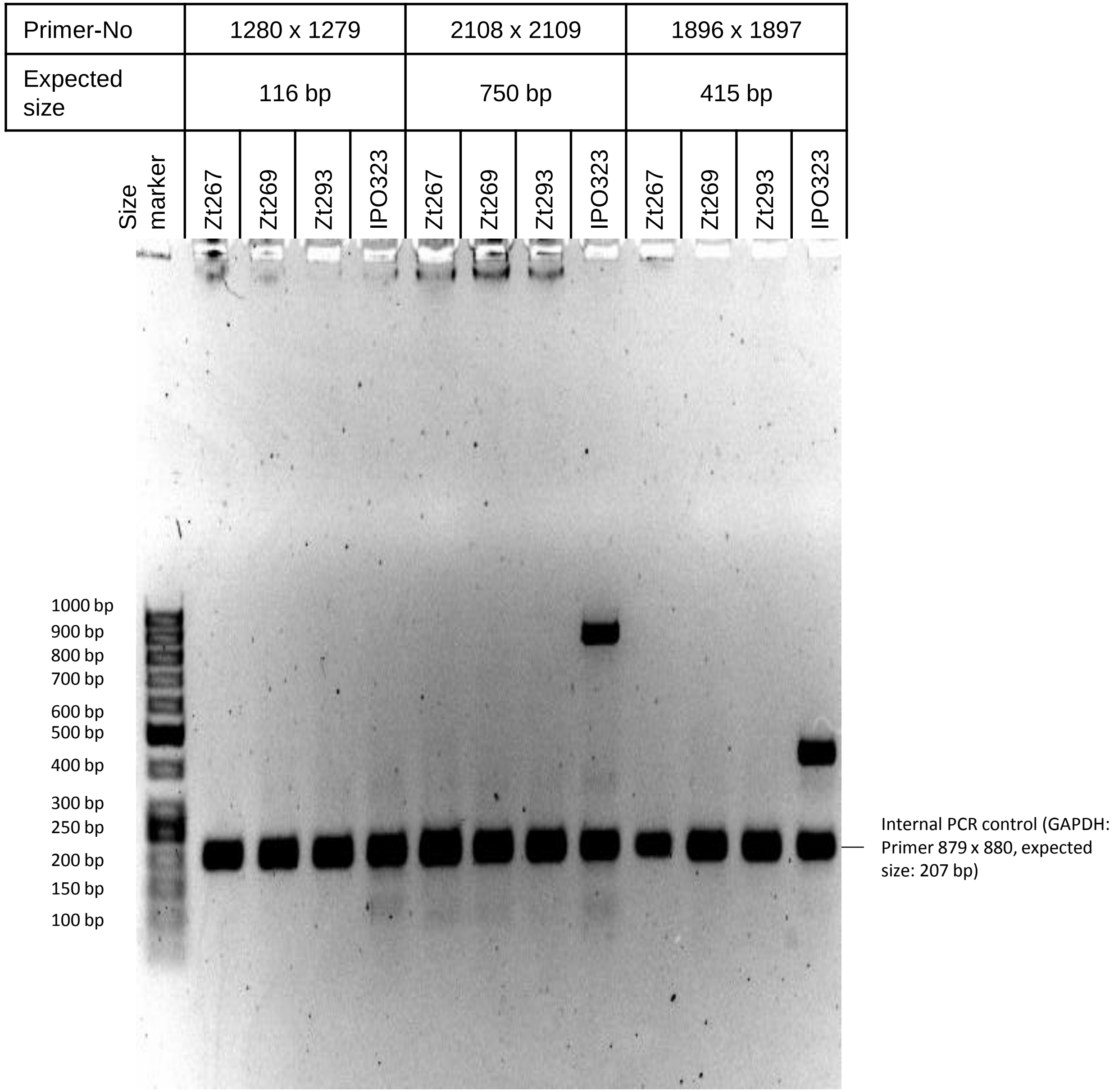

FIG S1 I Gel electrophoresis of PCR along the chromosome 21 for strains that had lost chromosome 21.
