# Supplementary material for: Forward Genetics Approach Reveals Host Genotype-Dependent Importance of Accessory Chromosomes in the Fungal Wheat Pathogen Zymoseptoria tritici
Source: mBio. 2017 Nov 28;8(6):e01919-17. doi: 10.1128/mBio.01919-17 (PMC5705923; doi:10.1128/mBio.01919-17)
Supplement: FIG S3 [file mbo006173611sf3.pdf]

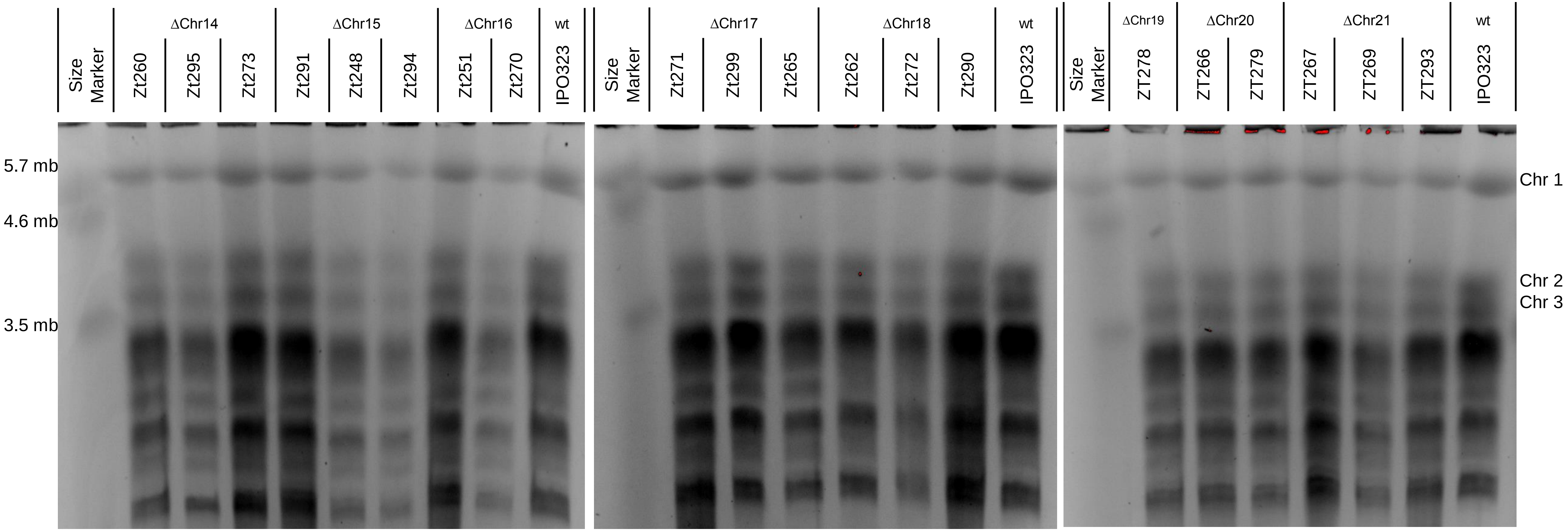

FIG S3 Pulsed-field gel electrophoresis for large core chromosomes of *Z. tritici*. Pictures of three pulsed-field gel electrophoresis gels for all strains used in this study. Size marker: *Schizosaccharomyces pombe* chromosomes
