# Supplementary material for: Forward Genetics Approach Reveals Host Genotype-Dependent Importance of Accessory Chromosomes in the Fungal Wheat Pathogen Zymoseptoria tritici
Source: mBio. 2017 Nov 28;8(6):e01919-17. doi: 10.1128/mBio.01919-17 (PMC5705923; doi:10.1128/mBio.01919-17)
Supplement: FIG S4 [file mbo006173611sf4.pdf]

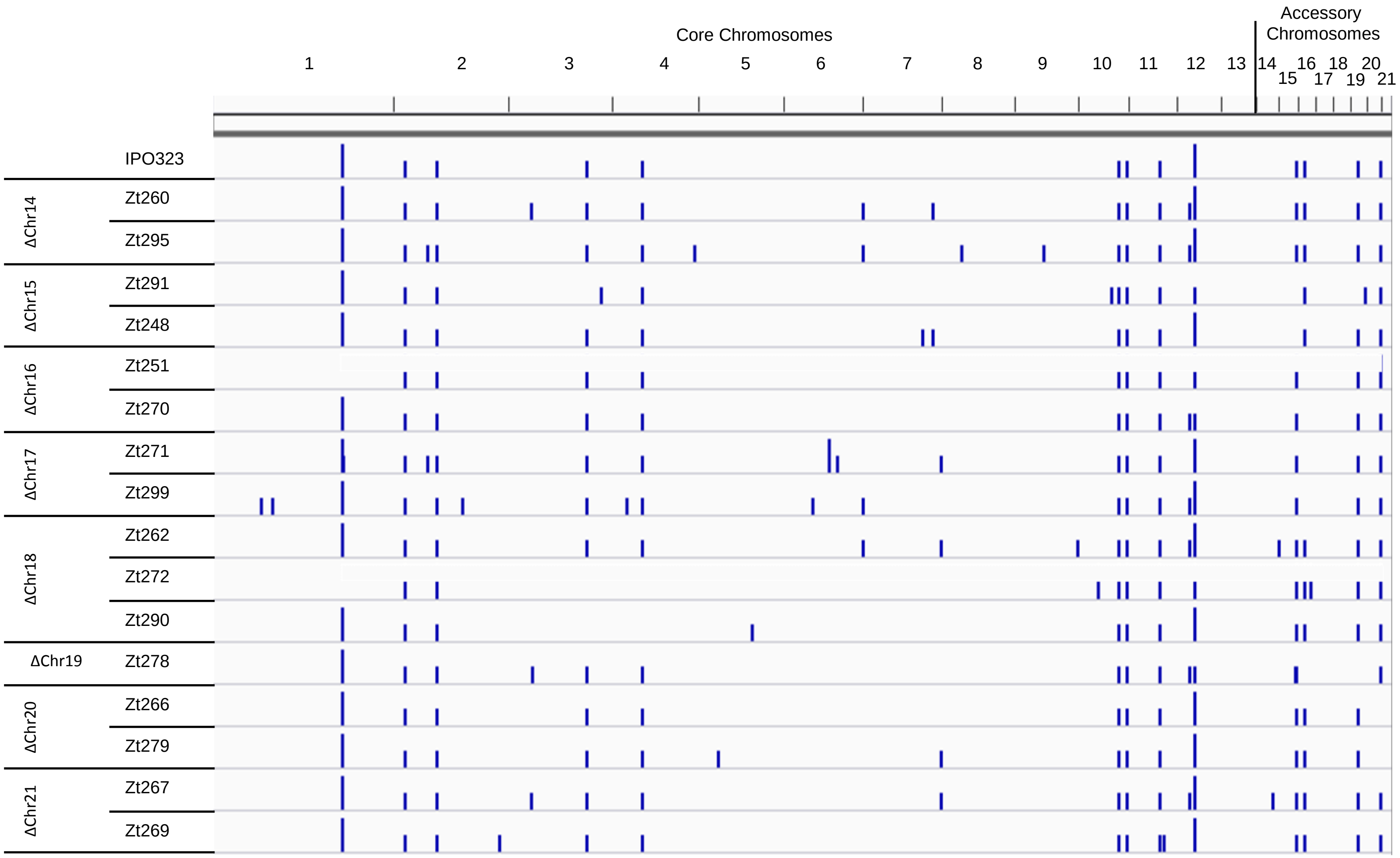

FIG S4 Illustration of the location of all single nucleotide polymorphisms (SNPs) and indels identified in all sequenced strains. Genome-wide distribution of SNPs across the thirteen core and eight accessory chromosomes of the reference strain IPO323.
