# Supplementary figures and images for: Forward Genetics Approach Reveals Host Genotype-Dependent Importance of Accessory Chromosomes in the Fungal Wheat Pathogen Zymoseptoria tritici
Source: mBio. 2017 Nov 28;8(6):e01919-17. doi: 10.1128/mBio.01919-17 (PMC5705923; doi:10.1128/mBio.01919-17)

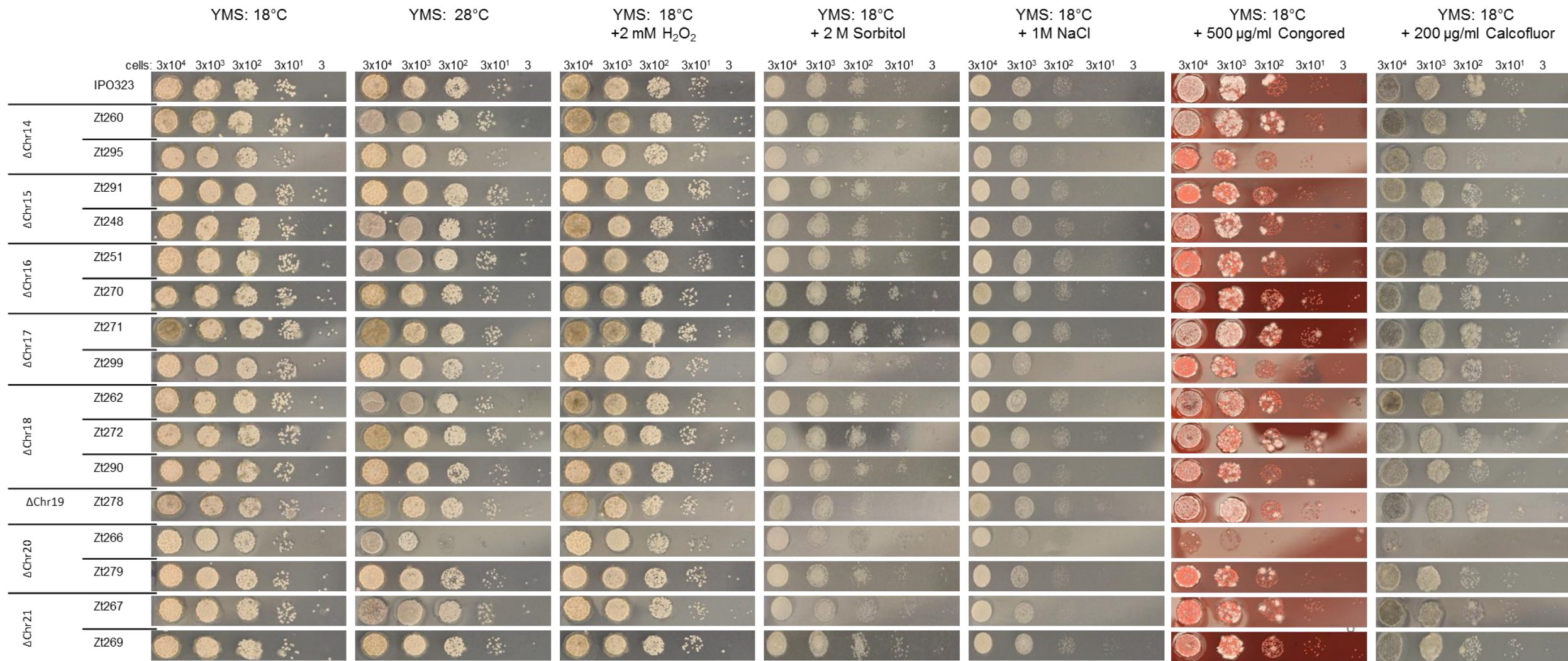

Supplement: FIG S5 [file mbo006173611sf5.pdf]
