# Supplementary material for: Forward Genetics Approach Reveals Host Genotype-Dependent Importance of Accessory Chromosomes in the Fungal Wheat Pathogen Zymoseptoria tritici
Source: mBio. 2017 Nov 28;8(6):e01919-17. doi: 10.1128/mBio.01919-17 (PMC5705923; doi:10.1128/mBio.01919-17)
Supplement: TEXT S1 [file mbo006173611s1.pdf]

Forward genetics approach reveals host-genotype dependent importance of accessory chromosomes in the fungal wheat pathogen *Zymoseptoria tritici*

Michael Habig, Jakob Quade and Eva Holtgrewe Stukenbrock

Supplementary text

Reference mapping of Illumina reads

Paired-end reads of 150 bp were mapped directly to the genome of the reference isolate IPO323 [1]. Processing of the reads were carried out using the below listed pipeline:

1) Quality filtering using Trimmomatic V0.30 [2]

   ```
   java -jar /trimmomatic-0.30.jar PE -phred33 R1.fastq R2.fastq R1_paired.fastq R1_unpaired.fastq R2_paired.fastq R2_unpaired.fastq HEADCROP:2 CROP:149 LEADING:3 TRAILING:3 SLIDINGWINDOW:4:15 MINLEN:50
   ```

   http://www.usadellab.org/cms/?page=trimmomatic

2) Mapping to IPO323 reference genome using Bowtie 2 version 2.1.0 [3]

   ```
   bowtie2 -p 6 -q -x IPO323_reference -1 R1_paired.fastq -2 R2_paired.fastq -S R.sam
   ```

   http://bowtie-bio.sourceforge.net/bowtie2/index.shtml

3) Converting to BAM and sorting using Picard 1.141

   ```
   java -jar /picard.jar SortSam INPUT=R.sam OUTPUT=R.bam SORT_ORDER=coordinate
   ```

   http://broadinstitute.github.io/picard

4) Removing duplicates using Picard 1.141

```
java -XX: +UseParallelGC -XX:ParallelGCThreads=6 -jar /picard.jar MarkDuplicates INPUT= R.bam OUTPUT=R_dedup.bam METRICS_FILE=metrics.txt REMOVE_DUPLICATES=true ASSUME_SORTED=true
```

5) Index Bamfile using samtools 0.1.19 [4]

```
samtools index R_dedup.bam
```

http://github.com/samtools/samtools

6) SNP calling using samtools 0.1.19 [4]

```
samtools mpileup -E -C50 -Q20 -q20 -uf IPO323.fa R_dedup.bam | ./bcftools call --ploidy-file ploidyfile -vc -O u -o R_dedup.bcf
```

7) BCF-VCF conversion using bcftools 0.1.19 [4]

```
bcftools view R_dedup.bcf > R_dedup.vcf
```

http://github.com/samtools/bcftools

8) Filtering using bcftools 0.1.19 [4]

```
bcftools filter -o R_filtered.vcf -s LOWQUAL -e'QUAL<20 | DP<10 | AF1<0.8' R_dedup.vcf
```

References

1. Goodwin SB, Ben M'Barek S, Dhillon B, Wittenberg, Alexander H. J., Crane CF, Hane JK, et al. Finished Genome of the Fungal Wheat Pathogen Mycosphaerella graminicola Reveals Dispensome Structure, Chromosome Plasticity, and Stealth Pathogenesis. PLoS Genet. 2011; 7: e1002070. doi: 10.1371/journal.pgen.1002070.
2. Bolger AM, Lohse M, Usadel B. Trimmomatic: a flexible trimmer for Illumina sequence data. Bioinformatics. 2014; 30: 2114–2120. doi: 10.1093/bioinformatics/btu170.
3. Langmead B, Salzberg SL. Fast gapped-read alignment with Bowtie 2. Nat Methods. 2012; 9: 357–359. doi: 10.1038/nmeth.1923.
4. Li H, Handsaker B, Wysoker A, Fennell T, Ruan J, Homer N, et al. The Sequence Alignment/Map format and SAMtools. Bioinformatics. 2009; 25: 2078–2079. doi: 10.1093/bioinformatics/btp352.
